# Stiffness anisotropy coordinates supracellular contractility driving long-range myotube-ECM alignment

**DOI:** 10.1126/sciadv.adn0235

**Published:** 2024-05-31

**Authors:** Nathaniel P. Skillin, Bruce E. Kirkpatrick, Katie M. Herbert, Benjamin R. Nelson, Grace K. Hach, Kemal Arda Günay, Ryan M. Khan, Frank W. DelRio, Timothy J. White, Kristi S. Anseth

**Affiliations:** ^1^ Department of Chemical and Biological Engineering, University of Colorado Boulder, Boulder, CO 80303, USA.; ^2^ The BioFrontiers Institute, University of Colorado Boulder, Boulder, CO 80303, USA.; ^3^ Medical Scientist Training Program, School of Medicine, University of Colorado Anschutz Medical Campus, Aurora, CO 80045, USA.; ^4^ Material, Physical, and Chemical Sciences Center, Sandia National Laboratories, Albuquerque, NM 87185, USA.

## Abstract

The ability of cells to organize into tissues with proper structure and function requires the effective coordination of proliferation, migration, polarization, and differentiation across length scales. Skeletal muscle is innately anisotropic; however, few biomaterials can emulate mechanical anisotropy to determine its influence on tissue patterning without introducing confounding topography. Here, we demonstrate that substrate stiffness anisotropy coordinates contractility-driven collective cellular dynamics resulting in C2C12 myotube alignment over millimeter-scale distances. When cultured on mechanically anisotropic liquid crystalline polymer networks (LCNs) lacking topography, C2C12 myoblasts collectively polarize in the stiffest direction. Cellular coordination is amplified through reciprocal cell-ECM dynamics that emerge during fusion, driving global myotube-ECM ordering. Conversely, myotube alignment was restricted to small local domains with no directional preference on mechanically isotropic LCNs of the same chemical formulation. These findings provide valuable insights for designing biomaterials that mimic anisotropic microenvironments and underscore the importance of stiffness anisotropy in orchestrating tissue morphogenesis.

## Teaser

Skeletal muscle cells collectively sense mechanical anisotropy of liquid crystalline networks and align in the stiffest direction.

## INTRODUCTION

Mechanotransduction and cell migration play critical roles in developmental, homeostatic, regenerative, and pathological biological processes (*1*, *2*). Early in vitro studies identified preferential cellular migration up a stiffness gradient using polyacrylamide hydrogels, a process termed durotaxis (*3*). Recently, Sunyer *et al.* (*4*) showed that while MCF10A cells did not durotax in isolation on polyacrylamide gels with a stiffness gradient >50 kPa/mm, clusters of cells migrated together toward stiffer gel regions in a form of collective cell durotaxis. The enhanced sensitivity of MCF10A monolayers to mechanical cues was found to be dependent on long-range force transmission through cell-cell junctions as well as cellular contractility, which effectively allowed cells to sense a stiffness gradient as a collective unit. Collective durotaxis has also been observed in vivo, where *Xenopus laevis* neural crest cells were found to follow a receding stiffness gradient during development, which enforced supracellular polarity of cell-matrix adhesions throughout the migrating cell cluster (*5*).

While cellular responses to stiffness gradients are relatively well understood, there is also strong precedent that stiffness anisotropy can serve as a mechanical cue influencing cellular behaviors such as polarization. Bischofs and Schwarz (*6*) introduced a connected theoretical framework to explain the observed polarization of single cells on anisotropic substrates, whereby focal adhesions (FAs) aligned in the stiffest direction mature more rapidly and outcompete FAs in other directions due to energetic favorability, causing cells to polarize with the stiffest direction of the substrate. This theory has been validated experimentally with isolated cells polarizing in the stiffest direction on strained collagen hydrogels as well as soft (~2 MPa) anisotropic polydimethylsiloxane micropillars (*7*, *8*). However, when the same cells were grown on anisotropic polystyrene micropillars with a much higher baseline elastic modulus (~2 GPa), no preferential cell polarization was observed (*7*). Thus, if a mechanically anisotropic substrate has sufficiently supraphysiological stiffness in all directions, the relative energetic favorability of FA maturation in the stiffest direction is diminished and isolated cells will polarize randomly (*6*). While single-cell responses to stiffness anisotropy may be limited by this mechanism, the boundaries of collective cellular responses to stiffness anisotropy have yet to be explored. This gap in understanding exists in part because there are limited strategies for generating mechanically anisotropic substrates that lack anisotropic topography (e.g., fibrous materials), which introduces confounding effects of contact guidance.

Liquid crystalline polymer networks (LCNs) are emerging as a unique class of anisotropic biomaterials for tissue engineering, medical devices, and soft robotics. Typical nematic liquid crystalline molecules (mesogens) contain a rigid rod-like aromatic core flanked by one or more reactive end groups (*9*). Molecular alignment of reactive mesogens can be enforced by surface, shear, and optical patterning methods due to strong intermolecular interactions and is retained upon crosslinking the mesogens into a network (*10*–*12*). As a result of the flexibility of approaches developed to program molecular anisotropy into a polymer network, there is growing interest in engineering LCNs to control tissue morphology. For example, Turiv *et al.* (*13*) used photoalignment techniques to synthesize LCNs with spatial control of ellipsoidal surface features, which guided fibroblast polarization to closely match the user-defined photopattern. Still, in vitro strategies that incorporate topographic features (e.g., fibrous materials) to investigate the (re)generation of aligned tissues cannot isolate the mechanobiological effects of stiffness anisotropy from those of contact guidance.

In cases where mesogen alignment is programmed to be unidirectional across a large area, polymerization of reactive end groups results in a monodomain LCN (mLCN) that is stiffest in the direction of molecular alignment, also known as the nematic director (*14*). Recently, fibroblasts cultured on mLCNs lacking topographic features were found to align with the nematic director (*15*). Notably, Martella *et al.* (*16*) also observed that C2C12 myoblasts cultured on mLCNs lacking topographic features aligned with the nematic director. However, the cellular dynamics involved in myoblast alignment were not addressed, and none of these studies have systematically explored whether changing the degree of substrate anisotropy affected alignment. LCN moduli and stiffness anisotropy can be tuned by altering the ratio of crosslinker to chain extender (*10*), thereby offering a flexible platform to probe the mechanoregulation of collective cellular responses to stiffness anisotropy in vitro.

Much like mLCNs, muscle tissue is inherently anisotropic with greater elastic modulus in the longitudinal direction compared to the transverse direction (*17*, *18*). It is well known that the highly anisotropic mechanics and architecture of the extracellular matrix (ECM) play critical roles in myoblast fusion and alignment during development and regeneration (*19*, *20*). However, the specific biophysical cues that direct myoblast polarization before fusion, as well as the collective cellular dynamics responsible for organizing aligned skeletal muscle tissue over large distances, remain unclear. We hypothesized that stiffness anisotropy acts as a mechanobiological cue for collective myoblast polarization and myotube alignment. To address this question, we cultured C2C12 murine myoblast cells on mLCNs and interrogated the collective cellular dynamics that emerged over increasing length scales.

Here, we demonstrate that C2C12 myoblasts cultured on mLCN substrates lacking topography adhere and polarize randomly until reaching a critical cell density threshold, after which supracellular coordination of actomyosin contractility develops, resulting in global myotube and ECM alignment with the nematic director over large distances (~millimeters). We found that myotube alignment was enhanced on stiffer mLCNs with greater orthogonal difference in elastic modulus and further scaled with the degree of stiffness anisotropy. Live-cell imaging and advanced analysis revealed that the combination of crowding dynamics, collective myoblast polarization, and contractile cellular flows mediated by reciprocal cell-ECM dynamics synergizes to yield long-range alignment of myotubes with the nematic director.

## RESULTS

### LCN films prepared by aza-Michael addition reaction exhibit distinct mechanical properties

**Fig. 1.**
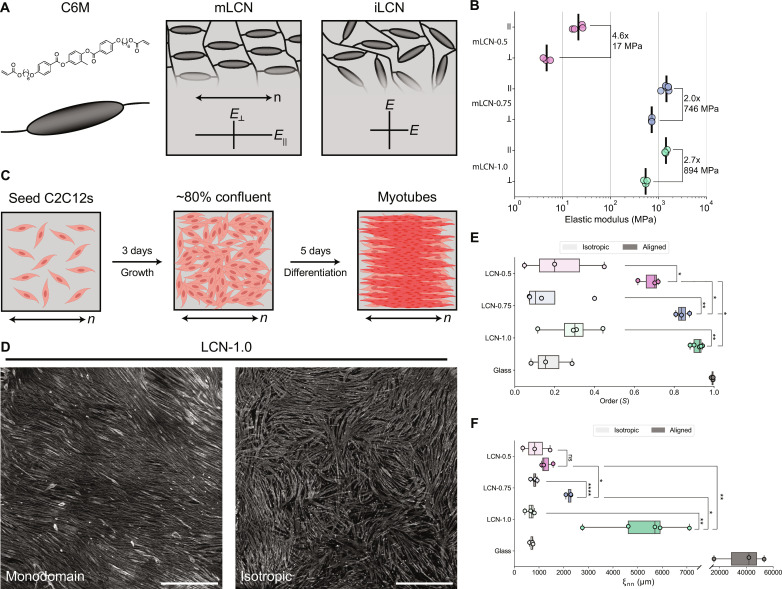
mLCN stiffness anisotropy drives C2C12 myotube ordering. (**A**) Illustration of mLCN and iLCN network structure and mechanical anisotropy (*n* indicates nematic director of mLCN). (**B**) Elastic modulus parallel and orthogonal to mLCN nematic director derived from the initial linear regime of stress-strain curves. Stiffness anisotropy ratio (*E*_||_/*E*_⊥_) and difference (*E*_||_ − *E*_⊥_) is calculated from the mean of repeated tensile tests. *N* = 4, 3, 6, 3, 3, and 3 (top to bottom); line indicates mean, all replicates shown. (**C**) Illustration of C2C12 growth and differentiation on mLCNs. Created with BioRender.com. (**D**) Representative images of myotubes stained for myosin II heavy chain (MF-20, gray) after 5 days of differentiation on monodomain (left) and isotropic (right) LCN-1.0. Scale bars, 1000 μm. (**E**) Myotube orientation-order parameter (*S*) and (**F**) nematic correlation length (μm) of myotubes after 5 days of differentiation on isotropic and aligned substrates (isotropic versus monodomain for LCNs, glass coverslip versus NanoSurface substrate for glass). *N* = 3, 3, 4, 3, 4, 5, 3, and 3 (top to bottom); the box limits extend from the 25th to 75th percentiles; the horizontal line indicates the median value; the whiskers extend by 1.5× the interquartile range; all replicates including outliers are shown. Statistical analysis was performed with one-tailed (isotropic versus monodomain) or two-tailed (monodomain versus monodomain) unpaired Student’s *t* test with Welch’s correction, with significance claimed at *P < 0.05, **P < 0.01, ***P < 0.001, ****P < 0.0001. ns, not significant.

LCNs are commonly prepared via photopolymerization of liquid crystalline monomers. Recent reports detail the utilization of chain-extending oligomerization reactions to adjust the glass transition temperature and associated moduli of LCNs (*10*, *11*). Motivated by the potential to study the collective cellular response of myoblasts to stiffness anisotropy, we focus on mLCNs prepared by surface-mediated alignment. Accordingly, we prepared mLCNs via an aza-Michael addition reaction at acrylate molar fractions of 0.5, 0.75, and 1.0 to fabricate cell culture substrates with orientational order over a range of moduli (Fig. 1A; fig. S1, A to C; and table S1). Isotropic LCNs (iLCNs) with the same chemical formulations but lacking stiffness anisotropy were prepared as controls. mLCN alignment was confirmed with polarized optical microscopy (fig. S1D). Tensile testing parallel and orthogonal to the nematic director was used to measure the magnitude of the elastic moduli as well as the degree of stiffness anisotropy between the three mLCN formulations (Fig. 1B and fig. S1E). iLCNs exhibit isotropic moduli in between the parallel and perpendicular elastic modulus for monodomain samples of the same formulation (*21*). As expected, the mLCN elastic moduli parallel to the nematic director trended with cross-link density (mLCN-0.5 < 0.75 to 1.0) (*10*). Meanwhile, the stiffness anisotropy ratio (*E*_||_/*E*_⊥_) was highest for mLCN-0.5 (4.6×), while the orthogonal difference in elastic modulus (*E*_||_ − *E*_⊥_) was highest for mLCN-1.0 (894 MPa). mLCN-0.75 provided a unique comparison to mLCN-1.0 as the elastic moduli parallel to the nematic director were almost identical (~1.4 GPa), while the degree of stiffness anisotropy was greater for mLCN-1.0 (Fig. 1B).

We then characterized the surface of mLCNs with atomic force microscopy (AFM) to assess for the presence of any topographic features that might confer contact guidance (fig. S1, G and H). All compositions had similar root mean square roughness measurements of ~2 nm. However, height maps of mLCN-0.75 and mLCN-1.0 indicated the presence of some nanoscale raised defects with a maximum height of ~10 nm, which were not anisotropic in their structure or distribution (fig. S1G). Many previous studies document the importance of the depth of topographical features in aligning cells, with a threshold of ~35 nm required for contact guidance (*22*, *23*). Therefore, the nanoscale features on these mLCNs were well below this threshold for imparting topographic guidance and are not expected to contribute to cell alignment. Finally, contact angle measurements showed little difference in surface energies between the three compositions, with all materials being slightly hydrophilic with contact angles ranging from 74° to 83° (fig. S1F).

### Myotube alignment is influenced by the degree of mLCN stiffness anisotropy

Following mLCN film preparation, we seeded C2C12 myoblasts at a relatively low density (5000 cells/cm^2^) on sterilized mLCNs as well as gelatin-coated glass coverslips for an additional isotropic, stiff control used in myoblast culture (*24*). Consistent with previous reports, we observed that C2C12s adhered to neat mLCNs, presumably due to nonspecific protein adsorption from the growth medium (*13*, *16*, *25*). Initially, we assessed whether isolated myoblasts would preferentially polarize with the nematic director. When myoblast orientation was quantified across the first 3 days of growth, there were no differences between mLCNs and glass coverslips (fig. S2, A and B). Furthermore, there were minimal differences among other measured cellular outputs (i.e., proliferation rate, cell area, and aspect ratio) before confluence (fig. S2, C to E). We attribute these similarities to the high overall stiffness (MPa to GPa) of all three mLCN compositions (*6*, *7*) and establish that both isolated myoblasts and small clusters of myoblasts cannot sense the stiffness anisotropy of these mLCNs.

After 3 days in growth medium, C2C12 myoblasts were ~80% confluent, at which point myoblast differentiation and fusion was induced by serum starvation (Fig. 1C) (*26*). Samples were fixed and immunostained after 3 and 5 days of differentiation, representing the stages of nascent myotube formation and myotube maturation, respectively (*27*, *28*). Prolonged culture times exceeding 6 days after the induction of differentiation were difficult to maintain, as myotubes became highly contractile and detached from their substrates, which has been previously observed in similar cultures (*26*). Myotube alignment (with a reference of 0° matching the orientation of the nematic director of the mLCN), two-dimensional (2D) orientation-order parameter (*S*, based on the 3D liquid crystalline nematic order parameter), and nematic correlation length (ξ_nn_) were quantified using OrientationJ and custom scripts (see Materials and Methods) (*24*, *29*).

After 3 days of differentiation, evidence of myotube alignment with the nematic director was already present on all three mLCN formulations (fig. S3). After 5 days of differentiation, myotubes cultured on mLCNs were highly aligned, whereas myotubes differentiated on iLCNs and glass displayed some regions of local alignment, but no long-range alignment (Fig. 1D and fig. S3A). Unexpectedly, the mean orientation of myotube alignment on mLCNs was consistently offset ~10° to 20° clockwise (CW) from the nematic director (figs. S3A and S4; Supplementary Text). Myotube order and ξ_nn_ increased between day 3 and day 5 on all mLCNs, while only ξ_nn_ increased on iLCNs and glass due to continued fusion and elongation of myotubes (fig. S3, B and C) (*24*). Notably, both myotube order and ξ_nn_ were significantly higher on all mLCNs compared to their formulation-matched iLCN controls, with the exception of ξ_nn_ for mLCN-0.5 (Fig. 1E). This direct comparison between C2C12 myotubes cultured on mLCNs and iLCNs is strong evidence that C2C12s collectively sense the stiffness anisotropy of mLCNs during differentiation and align with the stiffest direction. Furthermore, myotube order after 5 days of differentiation was significantly higher on the stiffer mLCNs [*S* = 0.84 (mLCN-0.75) and 0.92 (mLCN-1.0)] compared to the much softer mLCN-0.5 (*S* = 0.68) (Fig. 1E). Myotube order was also significantly greater on mLCN-1.0 compared to mLCN-0.75. Impressively, myotube order on mLCN-1.0 approached the order of myotubes grown on a commercial polymeric substrate with linear grooves used as a positive control for myotube alignment (NanoSurface, *S* = 0.99). The length scale of myotube alignment on mLCNs followed a similar trend with mean ξ_nn_ values of 1300, 2200, and 5200 μm for mLCN-0.5, mLCN-0.75, and mLCN-1.0, respectively (Fig. 1F).

### Stiffness anisotropy coordinates collective cellular dynamics across multiple length scales

**Fig. 2.**
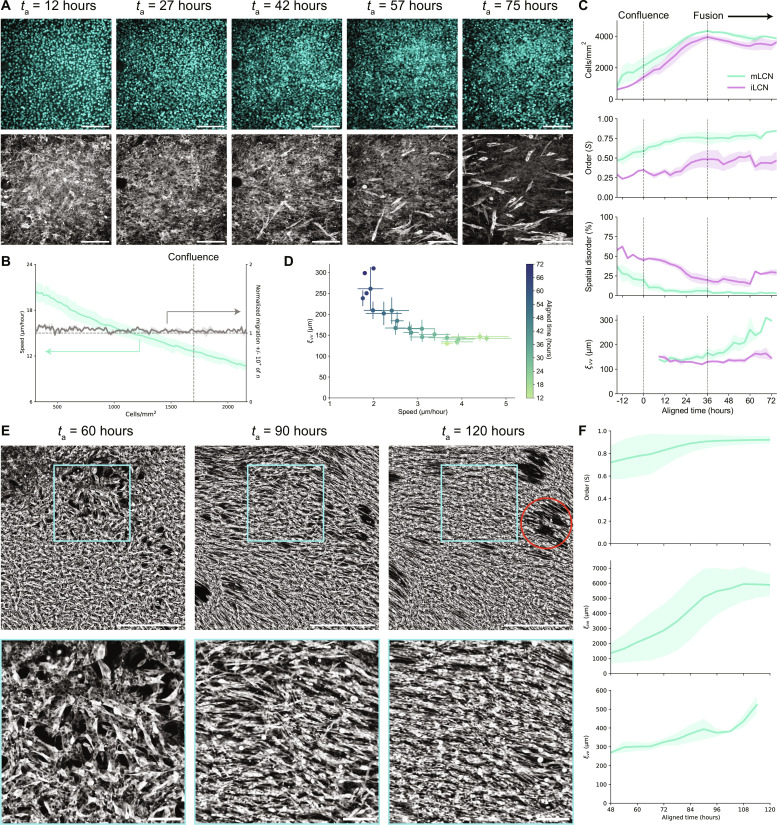
Stiffness anisotropy coordinates collective cellular dynamics across multiple length scales. (**A**) Representative images of C2C12 nuclei (top) and actin (bottom) on mLCNs at *t*_a_ = 12, 27, 42, 57, and 75 hours (left to right). Scale bars, 200 μm. (**B**) Myoblast migration speed (μm/hour; left axis) and normalized migration ±10° from nematic director (right axis) on mLCNs. Dashed line indicates approximate density at which myoblasts achieved confluence. *N* = 12 independent fields of view across three mLCNs; data are represented as means ± SEM. (**C**) Top to bottom: Temporal evolution of cell density (cells/mm^2^), orientation-order parameter (*S*), spatial disorder (%), and velocity correlation length (μm) on mLCNs and iLCNs. Dashed lines indicate approximate time at which myoblasts achieved confluence (left) and initiated fusion (right). *N* = 5 and 4 independent fields of view across three mLCNs and two iLCNs, respectively; data are represented as means ± SEM. (**D**) Velocity correlation length (μm) as a function of cellular speed (μm/hour) after confluence on mLCNs. *N* is the same as in (C); data are represented as means ± SEM. (**E**) Representative images (top) and inset (bottom) of C2C12 actin on mLCNs at *t*_a_ = 60, 90, and 120 hours (left to right). Scale bars, 1000 μm (top) and 200 μm (bottom). Red circle indicates location of a contractile defect. (**F**) Top to bottom: Temporal evolution of orientation-order parameter (*S*), nematic correlation length (μm), and velocity correlation length (μm) on mLCNs. *N* = 3 mLCNs; data are represented as means ± SEM.

To elucidate the spatiotemporal dynamics of myotube alignment on mLCNs, we used live-cell imaging with fluorescent DNA and actin probes and assessed the evolution of myoblast and myotube ordering. We primarily focused on LCN-1.0 as the anisotropic properties of mLCN-1.0 had the greatest effect on myotube alignment. Hereafter, these substrates are simply referred to as mLCNs and iLCNs. While we already established that single cells and clusters of cells did not align with the nematic director on mLCNs, we hypothesized that alignment could be induced by preferential migration of myoblasts in line with the nematic director above a critical cell density threshold. Although we characterized only the parallel and orthogonal mLCN modulus values, the mechanical properties are better described as a radial stiffness gradient (*30*, *31*), analogous to the linear stiffness gradient of the polyacrylamide gels used by Sunyer *et al.* (*4*) that induced collective durotaxis. Myoblast migration was evaluated at the single-cell level with nuclear tracking until the cell density became too high for accurate segmentation (>2200 cells/mm^2^). As expected, migration speed continuously decreased during the proliferative phase, as mobility became increasingly limited due to cellular crowding (Fig. 2B) (*32*). However, we found no strong evidence of orientationally biased C2C12 division (fig. S5) or preferential migration (Fig. 2B).

We then performed another set of live-cell imaging experiments on both mLCNs and iLCNs starting at ~80% confluence and ending after fusion of nascent myotubes (Fig. 2A and movie S1). Because of small experimental variation in initial seeding density, individual time series were aligned to each other in time (aligned time, *t*_a_) based on the time point at which myoblasts became confluent (*t*_a_ ~ 0 hours; see Materials and Methods). Myoblasts continued proliferating for an additional 36 hours past confluence, forming a multilayered culture with a maximum cell density of ~4000 cells/mm^2^ on both mLCNs and iLCNs (Fig. 2C) (*33*). Myoblast order on both substrates was low (*S* ≤ 0.5) at the start of imaging, complementing our previous analysis that found no preferential polarization of myoblasts with the nematic director after 3 days of growth (Fig. 2C and fig. S2B). However, we found that myoblasts collectively polarized with the stiffest direction of mLCNs as they approached confluence and continued proliferating (Fig. 2A and movie S1). Once myoblasts reached confluence at *t*_a_ = 0 hours, order on mLCNs had increased to 0.59 and continued to rise to 0.75 through *t*_a_ = 36 hours as myoblasts collectively polarized with the nematic director (Fig. 2C). Meanwhile, order remained below 0.5 on iLCNs through *t*_a_ = 36 hours. Spatial disorder, which was calculated as the percent of local regions (~50 × 50 μm) with an order parameter less than 0.5, decreased from 37% to just 6% on mLCNs from *t*_a_ = −15 to 36 hours. In contrast, spatial disorder remained above 20% at *t*_a_ = 36 hours on iLCNs (Fig. 2C). Therefore, we posit that myoblasts are able to collectively sense the stiffness anisotropy of the mLCN above a critical cell density threshold that allows them to coordinate cytoskeletal contractility and gradually polarize with the stiffest direction of the mLCN.

Next, we investigated whether this collective polarization was caused by collective C2C12 migration after confluence using particle image velocimetry (PIV) analysis of the actin signal (movie S1). Akin to the nematic correlation length (ξ_nn_), we calculated the velocity correlation length (ξ_vv_) from the angle of velocity vectors as a measure of the average length scale of collective migration. ξ_vv_ remained below ~150 μm on both substrates from *t*_a_ = 9 hours until the end of the proliferative phase at *t*_a_ = 36 hours, confirming that the collective myoblast polarization seen on mLCNs was not caused by preferential cellular migration (Fig. 2C). Proliferation was then arrested as myoblasts began to fuse, forming nascent multinucleated myotubes. Over the next 39 hours (*t*_a_ = 36 to 75 hours), a collective cellular flow polarized with the nematic director was evident on mLCNs, corresponding to an increase in ξ_vv_ from ~150 μm to ~300 μm (Fig. 2C and movie S1). C2C12 order increased from 0.75 to 0.85, and spatial disorder fell to just 2.8% on mLCNs from *t*_a_ = 36 to 75 hours as cellular alignment was further enhanced by collective cellular migration. In contrast to epithelial monolayers that undergo jamming transitions resulting in glassy dynamics with low velocity correlation lengths (*32*), C2C12 velocity correlation length increased with decreasing speed over time (Fig. 2D). While cellular flows also emerged on iLCNs during fusion, they were not polarized in any single direction, and thus, ξ_vv_ did not substantially increase. C2C12 order on iLCNs fluctuated around 0.5 during these randomly oriented cellular flows on iLCNs, yet spatial disorder increased from 20% to 30%, illustrating that randomly oriented cellular flows impede the development of global cellular alignment (Fig. 2C). In sum, collective myoblast polarization in the stiffest direction of mLCNs precedes emergent collective cellular flows, which serve to further homogenize cellular alignment after collective polarization.

Examining the time series more closely, most of the nascent myotubes arising from myoblast fusion are already polarized with the nematic director on mLCNs (Fig. 2A and movie S1). This might be expected as myoblasts were already polarized before fusion began and have a well-documented preference for anisotropic fusion (*27*). However, we also observed that nascent myotubes that were not initially polarized with the nematic director became increasingly aligned by the polarized flow field. To further elucidate the size scale of these collective cellular flows, we performed a similar experiment but imaged live cells with the fluorescent actin probe across the entire ~3 × 3 mm mLCN from *t*_a_ = 48 to 120 hours (Fig. 2E and movie S2). Using the same sequence of experiments and analysis routines, we again found that myoblast order was already high (*S* ~ 0.7) before the cellular flows began (Fig. 2F). As nascent myotubes arose, local 2D orientation-order parameter analysis identified the presence of a few disordered regions of myotubes highlighted by the presence of +^1^/_2_ and −^1^/_2_ topological defects (fig. S6 and movie S2). As the length scale of the collective cellular flows increased from ξ_vv_ = ~270 μm to ~520 μm (Fig. 2F), these topological defects annihilated and disordered myotubes were reoriented by the flow field to generate a highly ordered myotube culture (*S* = 0.92) that matched our results for myotube order in Fig. 1E (Fig. 2F, fig. S6, and movie S2). Meanwhile, ξ_nn_ increased from 1800 to 6000 μm, illustrating the marked effect of the collective cellular flows inducing global myotube alignment (Fig. 2F). Together, these results indicate that in the absence of stiffness anisotropy, differentiating myoblasts cannot coordinate myoblast polarization or the collective cellular flows that arise during fusion, leading to polydomain myotube alignment. In the presence of stiffness anisotropy, even when it is indistinguishable to single cells, myoblasts collectively polarize in the direction of greater stiffness. Subsequent collective cellular flows remain polarized with the nematic director and drive global myotube alignment on mLCNs.

### Reciprocal cell-ECM dynamics mediate collective cellular flows

**Fig. 3.**
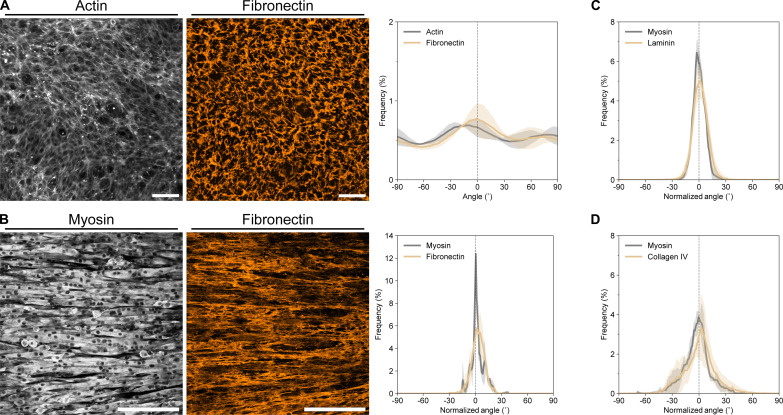
Nascent ECM alignment develops in parallel with myotube alignment. (**A**) Representative maximum intensity projections of actin (left) and fibronectin (middle) on mLCNs after reaching confluence. Scale bars, 100 μm. Corresponding frequency distribution plot (right) of actin and fibronectin alignment. *N* = 3 mLCNs; data are represented as means ± SD. (**B**) Representative maximum intensity projections of myosin II heavy chain (left) and fibronectin (middle) on mLCNs after 5 days of differentiation. Scale bars, 200 μm. Corresponding frequency distribution plot (right) of myosin and fibronectin alignment. *N* = 2 mLCNs; data are represented as means ± SD. (**C** and **D**) Frequency distribution plots of myosin and (C) laminin, or (D) collagen IV alignment after 5 days of differentiation. *N* = 5 mLCNs for (C) and 3 mLCNs for (D); data are represented as means ± SD.

To this point, we have established that collective myoblast polarization plays a role in templating the collective cellular flows before the onset of differentiation on mLCNs. However, our observations and previous in vitro studies have demonstrated that fused myotubes lie on top of a layer of myoblasts (*34*). Thus, nascent myotubes may not sense the stiffness anisotropy of the substrate, alluding to the contribution of an additional collective cellular dynamic. Given the importance of the ECM in muscle development and regeneration, we assessed whether dynamic cell-ECM interactions were mediating the increasing scale of these collective cellular flows. We first fixed and stained myoblasts grown on mLCNs shortly after they achieved confluence and found that a substantial amount of nascent fibronectin had already been deposited (Fig. 3A). Fibronectin alignment was quantified using OrientationJ, which revealed modest alignment with the nematic director that mirrored the myoblast actin alignment (Fig. 3A). This finding suggests that myoblasts are actively remodeling the ECM during the process of collective polarization. Subsequently, we examined the architecture of three key ECM proteins present in muscle tissue (*35*)—fibronectin, laminin, and collagen IV—after 5 days of differentiation on mLCNs. The alignment of all three cell-secreted ECM proteins was dramatically higher compared to the fibronectin staining before fusion and closely matched the corresponding myotube alignment in both magnitude and direction (Fig. 3, B to D and fig. S7). These results demonstrate that both cell and ECM alignment evolve together over time, but whether these cell-ECM dynamics mediate the collective cellular flows was still unclear.

Contractile cells are known to locally align their ECM, which in turn supports greater forces and amplifying anisotropic tension through the ECM that can align cells over large distances (*36*, *37*). In one report on these reciprocal cell-ECM dynamics, increasing fibroblast contractility exceeded the tensile strength of the nascent ECM, driving the eventual formation of contractile instabilities (*38*). Myotubes are known to become highly contractile, with a nascent myotube doublet exerting more than twice the summed force of two unfused myoblasts (*39*). Toward the end of our live-cell imaging experiments, we observed similar instabilities as highly contractile myotubes overpowered the ECM and created holes in the culture (Fig. 2E and movie S2). These holes developed along the axis of the collective cellular flows, with a slight CW offset to the nematic director in concordance with the offset myotube alignment in fig. S3. While the ECM is known to be viscoelastic on short timescales, it also exhibits plasticity when forces are applied over longer timescales (*40*). Therefore, we hypothesized that increasingly contractile myotubes exert coordinated, persistent forces on the ECM, causing plastic deformation and reorienting/displacing myotubes attached to and enmeshed within it.

**Fig. 4.**
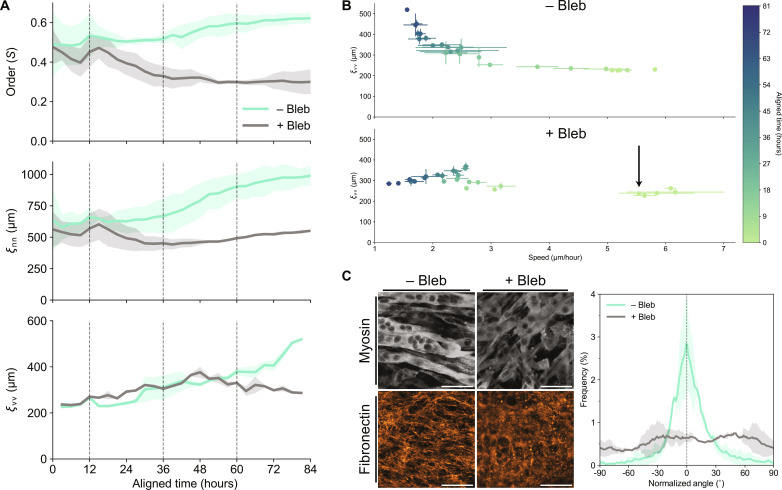
Reciprocal cell-ECM dynamics mediate collective cellular flows on mLCNs. (**A**) Top to bottom: Temporal evolution of orientation-order parameter (S), nematic correlation length (μm), and velocity correlation length (μm) on mLCN ± blebbistatin treatment. Dashed lines indicate timing of daily medium changes, with the first addition of blebbistatin occurring at *t*_a_ = 12 hours. *N* = 2 mLCNs for each condition; data are represented as means ± SEM. (**B**) Velocity correlation length (μm) as a function of cellular speed (μm/hour) on mLCN ± blebbistatin treatment. *N* is the same as in (A); data are represented as means ± SEM. Arrow indicates first addition of blebbistatin. (**C**) Representative maximum intensity projections (left) of myosin II heavy chain (top) and fibronectin (bottom) after 3.5 days of differentiation on mLCN ± blebbistatin treatment. Scale bars, 50 μm. Corresponding frequency distribution plot (right) of fibronectin alignment after 3.5 days of differentiation on mLCN ± blebbistatin treatment. *N* is the same as in (A); data are represented as means ± SD.

Accordingly, we tested whether we could prevent global myotube-ECM alignment on mLCN-1.0 by disrupting the positive feedback loop between increasingly contractile myotubes and the viscoplastic ECM. We treated C2C12s on mLCNs with the nonmuscle myosin II inhibitor blebbistatin daily for 3 days, beginning at confluence (*t*_a_ = 0 hours) and imaging every 3 hours (movie S3). Shortly after the addition of 10 μM blebbistatin, C2C12 order decreased from 0.45 to 0.3, with a corresponding decrease in ξ_nn_ from 600 μm to 450 μm (Fig. 4A). Blebbistatin had a delayed effect on ξ_vv_, which continued to increase in tandem with the control samples until *t*_a_ = 42 hours when it diverged and returned to the baseline velocity correlation length of ~240 μm. We attribute this delayed effect to the moderate ECM alignment found at confluence, which may provide mechanical memory for cellular migration (*41*). Meanwhile, C2C12 order, ξ_nn_, and ξ_vv_ increased as expected for samples not treated with blebbistatin (Fig. 4A). While the characteristic increase in ξ_vv_ with decreasing speed over time was again observed on uninhibited samples, the opposite trend was seen with blebbistatin treatment (Fig. 4B).

Finally, we immunostained these samples for myosin and fibronectin to assess the effect of disrupting cellular contractility on myotube fusion and ECM organization. Although myoblasts were still capable of differentiation and fusion in the presence of blebbistatin, the resulting myotubes were disordered and had fewer nuclei compared to untreated samples (Fig. 4C and fig. S8). We again found dense, aligned fibronectin on untreated samples, whereas on blebbistatin-treated samples, fibronectin staining was sparse and completely isotropic (Fig. 4C). These results demonstrate that coordinated supracellular contractility, which was disrupted with blebbistatin, is necessary for the emergence of contractile cell-ECM flows that drive global myotube-ECM alignment with the nematic director of mLCNs.

## DISCUSSION

### Stiffness anisotropy is a mechanobiological cue for collective cell-ECM alignment

Engineered biomaterials are increasingly used to study and direct biophysical cellular processes that give rise to the organized multicellular morphologies found in tissues and organs (*42*). This work provides an integrated analysis of how anisotropy can be transmitted and amplified across length scales from the liquid crystalline mesogen (~10 Å) of mLCNs to skeletal muscle myotube cultures spanning several millimeters. Molecular anisotropy of liquid crystalline mesogens is translated to mechanical anisotropy in the resulting polymer network, which serves as a mechanobiological cue to C2C12s enabling them to coordinate contractility in the stiffest direction (i.e., with the nematic director), resulting in global myotube-ECM alignment on the mLCN substrate. In the absence of anisotropy (i.e., when LCNs are polymerized in the isotropic state), myotubes do not develop long-range order, with 2D order and ξ_nn_ similar to that of myotubes grown on glass coverslips (Fig. 1 and fig. S3). Since the mLCNs had minimal topographic features, well below the requirement for contact guidance, we conclude that C2C12s are able to collectively sense the stiffness anisotropy of mLCNs.

Our use of an aza-Michael addition chemistry enabled us to systematically investigate how the degree of stiffness anisotropy in three different mLCNs affects myotube alignment without any confounding effects from topographic contact guidance. Although mLCN-0.5 displayed the greatest stiffness anisotropy ratio (*E*_||_/*E*_⊥_), myotube order and ξ_nn_ was higher on mLCN-0.75 and mLCN-1.0 (Fig. 1 and fig. S3), suggesting that myoblasts are sensing the orthogonal difference in elastic modulus (*E*_||_ − *E*_⊥_). Thrivikraman *et al.* (*43*) also found that the degree of fibroblast alignment on anisotropic fibrin gels scaled with the orthogonal difference in elastic modulus. However, the difference in overall stiffness between mLCN-0.5 and the two stiffer mLCNs cannot be overlooked and may contribute to the observed differences. We also found that myotube order and ξ_nn_ was significantly higher on mLCN-1.0 compared to mLCN-0.75. As the only difference between these formulations is a greater degree of stiffness anisotropy for mLCN-1.0, this comparison demonstrates that collective cellular alignment scales with the degree of stiffness anisotropy.

**Fig. 5.**
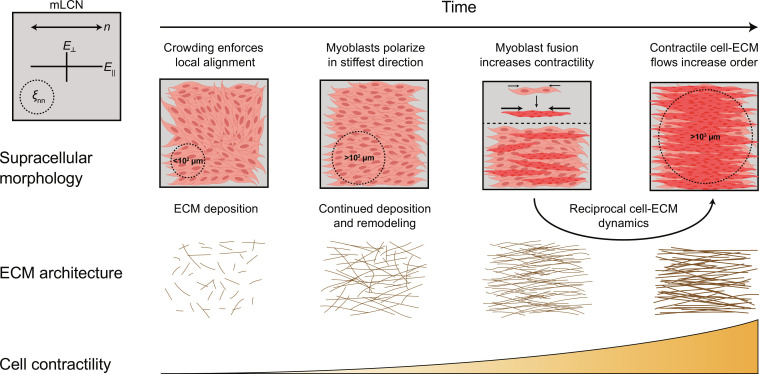
Spatiotemporal evolution of collective cellular dynamics that drive C2C12 myoblast and myotube alignment with the nematic director of mLCNs. Created with BioRender.com.

Through live-cell imaging and advanced image analysis, we identified three distinct temporal phases through which C2C12s interact with the mLCN substrate, neighboring cells, and nascently deposited ECM over increasing length scales to generate long-range order on mLCNs: (i) cellular crowding, (ii) collective myoblast polarization, and (iii) contractile cell-ECM flows mediated by reciprocal cell-ECM dynamics (Fig. 5). Across all three mLCN formulations, stiffness anisotropy had no effect on myoblast polarization or migration before confluence (fig. S2). This result was not unexpected, given multiple reports of single cells being unresponsive to stiffness anisotropy on substrates with high baseline stiffness (*6*, *7*, *44*). However, myoblasts exhibited enhanced sensitivity to stiffness anisotropy with increasing cell density, similar to the collective durotaxis of cell monolayers cultured on hydrogels with linear stiffness gradients that were undetectable by single cells (*4*). While epithelial monolayers undergo jamming transitions upon reaching confluence (*32*, *45*), we found that C2C12 myoblasts continue proliferating after reaching confluence and form a distinct multilayered culture (Fig. 2, A to C) (*33*). During the proliferative phase, myoblast migration speed decreased and order rose as cell crowding induced nematic packing (*46*), leading to local domains of alignment highlighted by the presence of topological defects (*47*).

Myoblast order on mLCNs was higher at confluence compared to iLCNs (Fig. 2C), suggesting that myoblasts on mLCNs begin polarizing in the stiffer direction once a critical cell density threshold is reached. Collective polarization in migrating cellular monolayers has been observed previously, whereby cells reorient in the direction of greatest normal stress to minimize intercellular shear stress in a process termed plithotaxis (*46*). However, we observed myoblast reorientation before the emergence of collective cellular flows on mLCNs. Still, these processes may be related. As the network of cell-cell and cell-substrate contacts mature, myoblasts coordinate actomyosin contractility in the direction that they can generate the greatest stress (i.e., the nematic director of the mLCN) and polarize accordingly.

ECM deposition and remodeling before and during collective myoblast polarization resulted in a moderately aligned ECM architecture that both supports increased contractile forces and propagates these forces across large distances through reciprocal cell-ECM dynamics (*37*, *38*). Shortly after proliferation was arrested, myoblasts began fusing into nascent myotubes capable of generating exponentially greater contractile forces. These forces resulted in plastic deformation of the cell-secreted ECM and highly correlated collective cellular flows, as any myotubes embedded within and adhered to the matrix flowed with it. PIV analysis revealed increasing correlation length of these flows only on mLCNs (Fig. 2C), likely due to a combination of preferential anisotropic fusion along the axis of collective myoblast polarization, moderate ECM alignment before fusion, and the continued influence of substrate stiffness anisotropy on collective dynamics. These long-range contractile cell-ECM flows on mLCNs resulted in annihilation of most topological defects in the myotube cultures, reorienting nearly all myotubes in the direction of greatest stiffness (Fig. 2E, fig. S6, and movie S2). This positive feedback loop between increasing cellular contractility and ECM alignment ultimately results in remarkable global ordering of C2C12 myotubes on mLCNs across several millimeters. As further evidence of these processes, inhibiting C2C12 contractility with blebbistatin disrupted the coordination of actomyosin contractility on mLCNs. Blebbistatin affected both collective myoblast polarization and contractile cell-ECM flows, which ultimately abrogated long-range myotube and ECM alignment (Fig. 4).

### Stiffness anisotropy organizes active stresses to guide tissue morphogenesis

These findings offer a unique comparison to the biophysical evolution of other collective cellular dynamics such as the aforementioned jamming transitions in epithelial monolayers, which have been shown to be highly sensitive to the relative strength of cell-cell and cell-matrix contacts (*32*). For example, where we found that C2C12s with weak cell-cell adhesions exhibit increasing velocity correlation length as cell density increases and velocity correspondingly decreases (Fig. 2D), the exact opposite was observed in epithelial cells with strong cell-cell adhesions (*32*). Transient blebbistatin treatment in otherwise glassy epithelial monolayers increased long-range velocity correlation (*48*). Meanwhile, when C2C12s were treated with blebbistatin, we observed decreasing velocity correlation length with decreasing speed over time (Fig. 4B).

In a more direct comparison, fibroblast alignment with the nematic director of mLCNs was found to be driven primarily by directed cell division along the substrate’s nematic axis and associated extensile stresses that caused annihilation of topological defects in the monolayer (*15*). In particular, the authors found that increasing cell density drove a gradual transition from isotropic migration in single cells to the formation of “lanes” of cells with coherent velocity profiles and ultimately a jammed monolayer with long-range alignment with the nematic director. However, the role of the ECM in coordinating the extensile stresses that act to align the monolayer was not assessed. An earlier study also found that epithelial cells exhibited collective extensile behavior, while mesenchymal cells collectively behaved as a contractile system (*49*). When cell-cell adhesions were weakened by knocking out E-cadherin in epithelial cells, their collective behavior switched from extensile to contractile with a concomitant increase in cell-substrate traction forces. Conversely, when the ability of E-cadherin knockout cells to generate substantial traction forces was reduced, either by culture on a soft substrate or when contractility was inhibited with blebbistatin, the cellular collectives switched back to extensile behavior. Additionally, ECM-guided alignment of cardiomyocytes on patterned substrates showed an effectively linear relationship between cell density and orientational order (*50*). When these cells were treated with N-cadherin blocking antibody to disrupt cell-cell contacts, orientational order remained unchanged in confluent cultures. Thus, the balance between cell-cell and cell-substrate (or cell-matrix) interactions dictates the nature of active stresses that emerge once cells become confluent and behave as a collective. In our system, C2C12 collective dynamics are dominated by contractile mechanisms associated with cell-substrate (e.g., collective polarization in response to stiffness anisotropy) and cell-matrix (e.g., reciprocal cell-ECM dynamics) interactions. Remarkably, it appears that stiffness anisotropy of mLCNs can establish anisotropy of both contractile and extensile stresses to drive collective cellular alignment independent of cell identity. The development of nematic-like alignment in cellular collectives may be necessary for the emergence of higher-order nematic cellular interactions, such as the cholesteric-like chiral layering of aligned C2C12 myotubes observed on mLCNs.

Together, our multi-scale analyses and similar studies of collective cellular dynamics reveal the mechanisms by which cell populations are controlled by biophysical interactions to orchestrate distinct coordinated behaviors in development and regeneration (*51*–*53*). Investigations of these collective cellular dynamics in vitro necessitate the continued evolution and adoption of engineered biomaterials with tunable mechanical properties, highlighted by our use of LCNs in the present study. LCNs are uniquely amenable to spatial patterning of nematic alignment and thus stiffness anisotropy, as well as programmable stimuli-responsive deformations, creating opportunities to investigate the interplay between substrate mechanics, user-directed stresses, and the active stresses that emerge in cellular collectives.

### Limitations of the study

It is important to highlight that the overall moduli (MPa to GPa) of the materials studied here, as well as their large (MPa) orthogonal differences in moduli, are orders of magnitude greater than that of most soft tissues, including muscle (*54*). Furthermore, these surface-aligned mLCNs restrict studies of collective cellular dynamics to the 2D cell-material interface. While the C2C12 cell line provides a uniform and consistent model system to study cellular responses to stiffness anisotropy, these findings should be extended to primary and induced pluripotent stem cell–derived muscle stem cells that require soft substrates such as gelatin or Matrigel. Therefore, future studies will focus on the development of innovative anisotropic hydrogel networks capable of 3D cell encapsulation with physiologically relevant mechanical properties. The development of biomaterials that more faithfully recapitulate the anisotropic mechanical properties of tissues and their ECM will enable the field to probe intrinsic biophysical processes that are essential to developmental and regenerative processes. Further, the ability to understand and harness these dynamics to program multicellular morphology and function holds great promise for the regenerative biology, medicine, and engineering communities.

## MATERIALS AND METHODS

### C2C12 cell culture

C2C12 murine myoblasts (CRL-1772, American Type Culture Collection) were passaged and cultured on TCPS (Grenier) for use up to passage 15. The sex of this cell line is unknown. C2C12s were cultured at 37°C and 5% CO_2_ in sterile-filtered growth medium, which consisted of high-glucose Dulbecco’s modified Eagle’s medium (DMEM; Gibco) supplemented with 20% fetal bovine serum (FBS) (Gibco), 1× GlutaMAX (Gibco), 1 mM sodium pyruvate (Gibco), penicillin (50 U/ml) (Gibco), streptomycin (50 μg/ml) (Gibco), and amphotericin B (1 μg/ml) (Gibco). GlutaMAX and sodium pyruvate are added as supplemental carbon sources to satisfy the high energy demands of muscle cells.

### Synthesis of LCNs

Liquid crystalline monomer 1,4-bis-[4-(6-acryloyloxyhexyloxy)benzoyloxy]-2-methylbenzene (C6M; Wilshire Technologies), hexylamine (Sigma-Aldrich), thermal inhibitor 2,6-di-*tert*-butyl-4-methylphenol (BHT; Sigma-Aldrich), and photoinitiator Irgacure-651 (I-651, Sigma-Aldrich) were used. C6M, 2 wt % I-651, and 1 wt % BHT were added to a glass vial, melted at 100°C, and vortexed to ensure homogeneity. Hexylamine was added at 85°C to achieve the final acrylate molar fraction of 0.5 and 0.75 in LCN-0.5 and LCN-0.75, respectively. The mixture was again vortexed and drawn by capillary action into 50-μm-thick alignment cells at 85°C. The alignment cells were fabricated using Elvamide-coated glass slides that were rubbed with velvet and adhered in anti-parallel arrangement with a mixture of optical adhesive and 50-μm spacers. The filled alignment cells were held for 18 hours at 75°C to facilitate aza-Michael addition within the nematic phase. Oligomers were subsequently photopolymerized within the alignment cell at 75°C with 15 min of ultraviolet (UV) (365 nm) light at 10 mW/cm^2^. In the case of LCN-1.0, monomers were melted, mixed, and drawn into cells as described above, but proceeded directly to photopolymerization as no hexylamine was added. The cells were soaked in water and carefully opened to remove the polymerized films. iLCNs were synthesized in the same manner but polymerized in the isotropic state at 135°C in Elvamide-coated glass cells that were not rubbed.

### Polarized optical microscopy

Molecular alignment of mLCNs, or lack thereof for iLCNs, was confirmed by the presence or absence of birefringence using a Nikon Eclipse Ci-Pol with a 5× objective. LCNs were observed at 0° and 45° rotation between crossed polarizers.

### Tensile testing

mLCNs were cut into strips parallel and perpendicular to the nematic director. Tensile tests were performed using an RSA-G2 DMA (TA Instruments) at a constant rate of 5% strain/min until failure. The elastic modulus values reported in Fig. 1B are calculated from the slope of the initial linear stress/strain regime (see also fig. S1E).

### Contact angle measurements

Contact angle experiments were performed using a custom contact angle setup that included a syringe pump (Aitoserlea) and high-speed camera (Plugable). While imaging continuously with the camera, a small (~4 μl) droplet of deionized water was extruded through a 32-gauge needle (Nordson) driven by the syringe pump until the surface tension released the droplet onto the LCN. The contact angle was determined using the contact angle plugin for ImageJ assuming a spherical droplet.

### Atomic force microscopy

Surface roughness measurements were conducted on an Asylum MFP-3D AFM. LCNs were mounted on stainless-steel AFM pucks using double-sided tape. Rectangular Si cantilevers with reflective Al coatings on the detector side were used to enhance spatial resolution and laser reflectivity (Nanosensors PPP-NCHR cantilevers with a nominal spring constant *k*_c_ = 42 N/m and tip radius *R* = 7 nm). Intermittent-contact mode images of surface heights *z* were collected at a scan rate of 1 Hz. The images were taken with a 5 μm × 5 μm scan area and 512 scan points and lines, which translated to a ≈10-nm pixel size. Subsequently, the images were processed with first-order plane fit and flattening routines to account for sample tilt. Cross-sectional profiles of the *z* maps were amassed to enable quantitative comparisons between the various films; the profiles presented in fig. S1G were typical of data from other regions in each image. Similarly, the root mean square roughness values were reported for each image to evaluate any relationship between average topography and surface response.

### Substrate preparation for cell culture

LCNs were cut into nearly square rectangles (~5 × 4 mm) with either the long or short axis corresponding to the nematic director, depending on the experiment. LCNs were sterilized with 70% ethanol/H_2_O, placed into a 24-well plate, and washed with sterile phosphate-buffered saline (PBS). Glass coverslips (12 mm) were sterilized with 70% ethanol/H_2_O, placed into a 24-well plate, and washed with sterile PBS. NanoSurface dishes (35 mm) (Curi Bio) were sterilized with 70% ethanol/H_2_O and washed with sterile PBS. Three hundred microliters of 0.1% gelatin in water (STEMCELL Technologies) was added to the coverslips and NanoSurface dishes, which were incubated for >30 min at 37°C to allow the gelatin to cure. Excess gelatin solution was removed from coverslips and NanoSurface dishes before cell seeding.

### C2C12 seeding, growth, and differentiation

C2C12 myoblasts were trypsinized from TCPS and resuspended in fresh growth medium. Growth medium (1 ml) containing 5000 cells/cm^2^ were added to the wells with LCNs, gelatin-coated coverslips, or NanoSurface dishes. Cells were allowed to adhere to LCNs for 2 hours, at which time they were transferred to empty wells with 1 ml of growth medium to ensure that cells were only present on the LCNs. Cells were cultured in growth medium for 72 hours until ~70 to 80% confluent. At this point, growth medium was replaced with sterile-filtered differentiation medium to induce differentiation via serum starvation. Differentiation medium consisted of high-glucose DMEM (Gibco) supplemented with 5% horse serum (Gibco), penicillin (50 U/ml) (Gibco), 1× GlutaMAX (Gibco), 1 mM sodium pyruvate (Gibco), streptomycin (50 μg/ml) (Gibco), and 1× ITS Supplement (R&D Systems). Differentiation medium was replaced daily for up to 5 days, at which point cells were fixed.

### Immunofluorescence staining

All steps were carried out at room temperature. Samples were washed once with PBS and fixed with 4% paraformaldehyde (Electron Microscopy Science) diluted in PBS for 10 min. Samples were washed with PBS three times for 5 min each. Samples then were permeabilized and blocked with 0.1% Triton X-100 in PBS containing 3% bovine serum albumin (Sigma-Aldrich) for 30 min. Samples were incubated with primary antibodies diluted in the same buffer for 1 hour. The following primary antibodies were used: MF-20 (1:250, eBioscience, 14-6503-83), fibronectin (1:500, Abcam, ab2413), laminin (1:500, Abcam, ab11575), and collagen IV (1:500, Abcam, ab19808). After washing with PBS three times for 5 min each, samples were incubated with secondary antibodies in the same buffer as primary antibodies for 1 hour in the dark. The following secondary antibodies were used: Alexa Fluor 488 goat anti-mouse (1:250, Invitrogen, A-11001), Alexa Fluor 647 goat anti-rabbit (1:500, Invitrogen, A-21245), rhodamine phalloidin (1:500, Cytoskeleton, PHDR1), Alexa Fluor 488 phalloidin (1:500, Invitrogen, A12379), and 4′,6-diamidino-2-phenylindole (DAPI) (1 μg/ml, Sigma-Aldrich, 10236276001). Samples were washed with PBS three times for 5 min each before imaging.

### Fixed sample imaging

Fixed samples were imaged on multiple microscopes including a Zeiss LSM710 laser scanning confocal microscope equipped with 20×/1.0 NA (numerical aperture) water objective, a Nikon A1R laser scanning confocal microscope equipped with 10×/0.45 NA Air or 20×/0.95 NA long working distance water objective, or a Nikon Ti-E microscope equipped with 10×/0.45 NA and 20×/0.75 NA air objective, an Okolab environmental chamber, and a CREST X-Light V2 spinning disk system.

### Live-cell imaging

C2C12 myoblasts were seeded on LCNs as described above. Once cells were adhered, SiR-Actin (1:1000, Cytoskeleton, CY-SC001) and/or SPY-555-DNA (1:2000, Cytoskeleton, CY-SC201) was added to each well. Samples were imaged every ^1^/_3_, 1, 3, or 6 hours using a Nikon Ti-E microscope equipped with 10×/0.45 NA or 20×/0.75 NA air objective, an Okolab environmental chamber (37°C, 5% CO_2_, 95% relative humidity), and a CREST X-Light V2 spinning disk system.

### Image analysis

All image analysis was conducted in ImageJ except for PIV and topological defect detection conducted in MATLAB. Maximum intensity projections were used for most analysis. For myotubes cultured on mLCNs, the images were rotated such that the nematic director of the mLCN was parallel to the horizontal (0°) axis. For myotubes cultured on coverslips or iLCNs, the images were not rotated. For myotubes cultured on NanoSurface dishes, images were rotated such that the nanopatterned grooves were parallel to the horizontal (0°) axis. All images were cropped to exclude cells within 500 μm of the edges of the LCN, coverslips, and NanoSurface dishes due to contact-guidance effects of free edges. For the single-cell analysis in fig. S2, images were thresholded and segmented and ellipses were fit to individual cells. The number of cells, area, aspect ratio, and orientation were quantified with the Analyze Particles tool in ImageJ. Orientation counts were binned in 20° increments, converted to frequencies, and averaged across replicates. Cell density, area, and aspect ratio parameters were averaged for all cells in a single sample and time point and then averaged across replicates. For the intensity frequency plots in fig. S4, composite images were resliced to *XZ* projections without interpolation and summed across all *Y*. Then, average intensities of each z-slice were taken across all *X*. Intensity values were converted to frequencies, averaged across independent samples, and displayed as means ± SD. For the axis of myoblast division in fig. S5, individual frames of SPY-555-DNA–stained nuclei were examined and a line was manually drawn between the center of two nuclei of dividing cells. The orientation of these lines was taken with respect to the horizontal axis (i.e., nematic director), and counts were binned in 20° increments, converted to frequencies, and averaged.

### OrientationJ analysis

OrientationJ analysis (*29*) was performed on each image with σ = 10 or 20 pixels (for 10× and 20× objective, respectively) for myotube analysis and σ = 1 or 2 pixels (for 10× and 20× objective, respectively) for ECM analysis, with minimum coherency set to 25%. When the confocal microscope was used to acquire images with greater resolution (Fig. 4C and fig. S4), σ was set to the approximate width of myotubes or ECM fibrils in pixel units. Counts from the OrientationJ results were converted to frequencies. For ECM alignment analysis in Fig. 3 (B to D) and fig. S4, the orientation frequency distribution was shifted such that the angle of maximum myotube alignment for each sample was 0°. This was done due to the variation in the degree of myotube offset to the nematic director (fig. S3 and Supplementary Text). The frequency distribution of ECM orientation was then shifted by the same degree. When this strategy was used, the *x* axis reads “Normalized Angle (°).” Frequencies were then averaged and displayed as means ± SD. For the chiral layer analysis in fig. S4, maximum intensity projections of each layer were used and OrientationJ analysis was performed on each image as described above.

### Cell density and time series alignment

Time series images of SPY-555-DNA–stained nuclei were imported into ImageJ, and nuclei were detected using the StarDist plugin [model: versatile (fluorescent nuclei), probability threshold = 0.65, nmsThreshold = 0.55] (*55*, *56*). The spots were exported to the ROI manager, and the number of nuclei was determined by running the Analyze Particles tool. Cell density was calculated by dividing the number of cells at each time point by the area in mm^2^. Because of the dependence of cellular alignment on cell density and small experimental variation in initial cell seeding density causing large differences in cell density after several days of exponential growth, the start time of every time series was adjusted such that the maximum cellular density was achieved at *t*_a_ ~ 36 hours, which corresponded to cells achieving confluence at *t*_a_ ~ 0 hours. All other analyses (order, spatial disorder, ξ_vv_, speed) reported in Fig. 2 (C and D) were adjusted to the start times determined by this method. This resulted in some time points having just one value for cell density, order, spatial disorder, ξ_vv_, and speed, and thus, no standard error is reported for those points. Live-cell imaging for Fig. 2 (E and F) ended after 5 days of differentiation, and thus, the final time point was set as *t*_a_ = 120 hours. Live-cell imaging for Fig. 4 started once cells reached confluence and thus at *t*_a_ = 0 hours.

### Migration analysis

Time series images of SPY-555-DNA–stained nuclei were locally enhanced with the contrast-limited adaptive histogram equalization (CLAHE) tool (block size: 100; histogram bins: 256; maximum slope: 3) and imported into the ImageJ plugin TrackMate where nuclei were detected using the StarDist tracker (*55*, *56*). Nuclei with diameter larger than 30 μm or smaller than 6 μm were excluded. Tracks were created using the Simple LAP Tracker with default settings. Spots and Tracks were exported to a MotilityLab spreadsheet and imported into MATLAB where a custom script calculated mean speed and angle of migration. Cell density was calculated by dividing the number of cells at each time point by the area in mm^2^. The normalized cellular migration within ±10° of the nematic director was calculated by first obtaining the angle of migration of each cell at each time point, then calculating the summed frequency of cells falling into 2 bins (−10° to 0°, 0° to 10°), and dividing by the frequency for isotropic migration (2 bins out of 18 total = 11.111%). Mean migration speed and normalized migration within ±10° of the nematic director as a function of cell density was obtained by averaging linear interpolations of individual migration analyses with increments of 10 cells/mm^2^.

### PIV analysis

PIV analysis was conducted using the PIVlab version 2.61 software plugin for MATLAB. For all PIV analysis, image preprocessing parameters included CLAHE: 64 pixels and auto contrast stretch. PIV settings for Fig. 2 (C and D) were as follows: pass 1: 300-pixel interrogation area/150-pixel step size, pass 2: 150-pixel interrogation area/75-pixel step size. PIV settings for Fig. 2F were as follows: pass 1: 600-pixel interrogation area/300-pixel step size, pass 2: 300-pixel interrogation area/150-pixel step size, pass 3: 150-pixel interrogation area/75-pixel step size. PIV settings for Fig. 4 (A and B) were as follows: pass 1: 1000-pixel interrogation area/500-pixel step size, pass 2: 500-pixel interrogation area/250-pixel step size, pass 3: 250-pixel interrogation area/125-pixel step size. Vectors were smoothed at the lowest setting, and aberrant vectors at the frame boundaries were manually rejected to remove artifacts. Velocity magnitudes were averaged across each frame and then across independent samples and converted to μm/hour. After medium changes, the field of view tended to shift slightly, resulting in artificially high autocorrelation at certain time points. While others have subtracted the mean velocity at each time point to eliminate this artifact, this is only applicable for isotropic systems (*32*). In our system, the flows are polarized, and thus, we chose to remove those artifactual data points from our analysis altogether. These frames are dimmed in movies S1 to S3, which display autoscaled vectors overlaid on a heatmap of velocity magnitude ranging from 0 to 5 μm/hour (movie S1), 0 to 8 μm/hour (movie S2), and 0 to 7 μm/hour (movie S3).

### Topological defect detection using line integral convolution (LIC)

OrientationJ VectorField was run on images of 2500 × 2500 pixels with σ = 10 pixels and grid size = 5 pixels. Resulting .csv files were imported into Python (version 3.9) where the “lic” package (version 0.4.5) was used to create the images in movie S2 with the “length” variable set to 20. For fig. S6, the original LIC plots were enhanced by running the LIC script again with the seed image set to the original LIC image, followed by histogram equalization with a power of 1.5 to further enhance contrast. Images were cropped and imported into MATLAB where the “Defector” code was run with tensorSize = 20, minDist = 30, and pixSize = 1 (*57*).

### Calculation of 2D orientation-order parameter (*S*)

Images were cropped into squares with pixel size a multiple of 100 (e.g., 1300 × 1300 pixels), and OrientationJ VectorField was run with σ = 10 or 20 pixels and grid size = 100 or 50 pixels (for 10× and 20× objective, respectively). 2D orientation-order parameter and nematic correlation length were calculated using custom R scripts adapted from Mao *et al.* (*24*). Order was calculated using the orientation of each vector with the following equation (*58*), where θ represents the difference between the angle of an individual vector and the mean of all angles in the image, and the brackets 〈〉 denote an average over grid points.

$$S=|\langle 2*{\text{cos}}^{2}\left(\theta \right)-1\rangle |$$ (1)

This equation provides the 2D orientation-order parameter in a range from 0 to 1, where 0 is completely isotropic and 1 is perfect order. For global order calculations, *S* was calculated over all angles in the image. For spatial disorder and local order calculations, *S* was calculated for 4 × 4 grids of vectors at each center position. Spatial disorder was calculated as the percentage of grids with an orientation-order parameter below a threshold of 0.5. For the grayscale order heatmap in movie S2, local orientation-order parameter (0 to 1) at each position was scaled to 8-bit grayscale values (0 to 255), imported into ImageJ as a text image, and saved as .tiff file.

### Calculation of nematic and velocity correlation length

Nematic correlation length (in pixel units) was taken as the *X* intercept of the least-squares regression of the first 10 points in the spatial autocorrelation function of the vector field output from OrientationJ:

$$C\left(d\right)=2*\langle {\text{cos}}^{2}\left[\theta \left(r\right)-\theta \;(r+d\right)]\rangle -1$$ (2)

where θ(*r*) is the local orientation angle of the vector field at grid position *r*, *d* is the separation vector to a second grid position *r* + *d* at distance |*d*| = *d*, and brackets 〈〉 denote an average over all pairs of grid points with the same distance *d*. Nematic correlation length was converted to μm by multiplying it with the pixel size (μm/pixel) of each image. For velocity correlation length calculations, vector angles from PIVlab were passed to a custom MATLAB script to format the data for import into R. The same script and equation (Eq. 2) used to calculate the nematic correlation length was used to calculate the velocity correlation length, again taking the *X* intercept of the least-squares regression of the first 10 points in the spatial autocorrelation function and multiplying it with the pixel size (μm/pixel) of each image to obtain the velocity correlation length in μm.

### Statistical analysis

Circular statistics were performed using a custom script with the package “circular” in R adapted from Davidson *et al.* (*59*). The Watson-Wheeler test for homogeneity was used to compare myotube alignment on mLCNs and iLCNs of the same formulation and time point. GraphPad Prism 9 was used for the remainder of statistical analysis. Statistical comparisons between two experimental groups were assessed via one-tailed or two-tailed unpaired Student’s *t* test with Welch’s correction. One-tailed *t* test was performed to compare orientation-order parameter and nematic correlation length of myotubes cultured on mLCNs versus iLCNs as the hypothesis was that cells would align better on mLCNs. Two-tailed *t* tests were used to compare the same measures between mLCN formulations as we had no preexisting hypothesis regarding the differences in myotube alignment between conditions.

## Supplementary Materials

### This PDF file includes:

Supplementary Text Figs. S1 to S8 Table S1 Legends for movies S1 to S3 References
