# Supplementary material for: Stiffness anisotropy coordinates supracellular contractility driving long-range myotube-ECM alignment
Source: Sci Adv. 2024 May 31;10(22):eadn0235. doi: 10.1126/sciadv.adn0235 (PMC11141631; doi:10.1126/sciadv.adn0235)
Supplement: Supplementary file 1 — Supplementary Text Figs. S1 to S8 Table S1 Legends for movies S1 to S3 References [file sciadv.adn0235_sm.pdf]

Science Advances
AAAS

# Supplementary Materials for

## Stiffness anisotropy coordinates supracellular contractility driving long-range myotube-ECM alignment

Nathaniel P. Skillin *et al.*

Corresponding author: Timothy J. White, tim.white@colorado.edu; Kristi S. Anseth, kristi.anseth@colorado.edu.

*Sci. Adv.* **10**, eadn0235 (2024)
DOI: 10.1126/sciadv.adn0235

**The PDF file includes:**

Supplementary Text
Figs. S1 to S8
Table S1
Legends for movies S1 to S3
References

**Other Supplementary Material for this manuscript includes the following:**

Movies S1 to S3

## Supplementary Text

### C2C12 myotubes develop chiral layering

When analyzing maximum intensity projections of myotubes grown on mLCNs, we observed that the dominant orientation was consistently offset in a clockwise (CW) direction from the nematic director **(Fig. S3A)**. The magnitude of this offset varied between ~10–20° after five days of differentiation on all mLCNs **(Fig. S4A)**. Prior reports have shown that C2C12 myoblasts demonstrate chiral orientation on fibronectin micropatterns due to chiral actin reorganization and cellular jamming (*60, 61*). When cultured in thin, rectangular fibronectin micropatterns, C2C12 myoblasts were found to exhibit a similar chiral offset (*62*). Uniquely, when we imaged myotube cultures on mLCNs after five days of differentiation with greater axial resolution, we identified distinct myotube layers with a chiral offset to the underlying layer **(Fig. S4B)**. The bottom layer, closest to the substrate surface, was typically aligned with the nematic director. The top layer was ~10° CW offset from the layer below **(Fig. S4C)**. We then considered whether this chiral layering was unique to mLCNs or could be replicated on the grooved NanoSurface substrate. Indeed, we found that a second layer of myotubes emerged on top of the layer in contact with the nanogrooves after five days of differentiation and exhibited a similar ~7° CW offset **(Fig. S4E and S4F)**. Thus, regardless of the mechanism of alignment (stiffness anisotropy vs. contact guidance), the inherent chirality of C2C12 myoblasts is retained upon fusion into myotubes and extends into the third dimension. This chiral myotube layering also explains why we observed a CW shift in the alignment frequency when analyzing the orientation of maximum intensity projections in **Fig. S3**.

Given the importance of reciprocal cell-ECM dynamics in driving global myotube alignment on mLCNs **(Fig. 3 and 4)**, we also assessed the ECM architecture with confocal microscopy. We found that laminin staining on mLCNs matched both the chiral twist and density of signal (i.e., fluorescence intensity) of the myotube layers (**Fig. S4B–D**). Laminin alignment on the NanoSurface substrate also matched the chiral twist of the myotubes. However, laminin was surprisingly sparse in the bottom layer of myotubes in contact with the grooves. Laminin density only increased in the top layer of myotubes above the nanogrooves **(Fig. S4E–G)**. This suggests that nascent ECM deposition and remodeling is required for the evolution of chiral myotube layers and maintenance of long-range alignment once myotubes escape contact with topographic features that enforce alignment. These findings also indicate that contact guidance-mediated myotube alignment occurs independent of reciprocal cell-ECM interactions, again highlighting the importance of reciprocal cell-ECM dynamics in maintaining long-range myotube alignment and chiral layering on mLCNs that lack topography.

While the evolution of chiral layers was not the focus of this study, we can still comment on some possible explanations and mechanisms. The chiral offset of C2C12s cultured on thin, rectangular fibronectin micropatterns was found to be a result of the emergence of a shear stress field due to appositional migration on opposing edges of the micropattern (*62*). However, this effect decayed with increasing width of the micropattern and did not result in global order for widths greater than 1000 μm, limiting translation of these results to our studies on mLCNs which were at minimum 3x3 mm. The most relevant study to date found that C2C12 myoblasts evolve into a multilayered culture at stationary +½ topological defects (*33*). In that report, the top layer develops a 90° offset to the bottom layer. Given the presence of multiple topological defects in our cultures before collective cellular flows emerge, it is feasible that these myoblasts initially develop the same 90° offset but are then reoriented to a final offset of ~10–20° during the collective cellular flows that align myotubes with the stiffest direction. In another study, C2C12 myoblasts cultured on circular fibronectin micropatterns of the same size scale as the nematic correlation length of

C2C12 myoblasts develop into 3D cellular mounds (*47*). These mounds were formed by a combination of active anisotropic stresses and collective cellular flows, with myoblasts exhibiting an increasing chiral offset to the micropattern boundary with each additional layer. Regardless of the mechanism of chiral layering in our study, it is certainly a result of the interplay between the active anisotropic stresses that govern collective cellular dynamics and dynamic cell-ECM interactions that maintain 3D multicellular morphology.

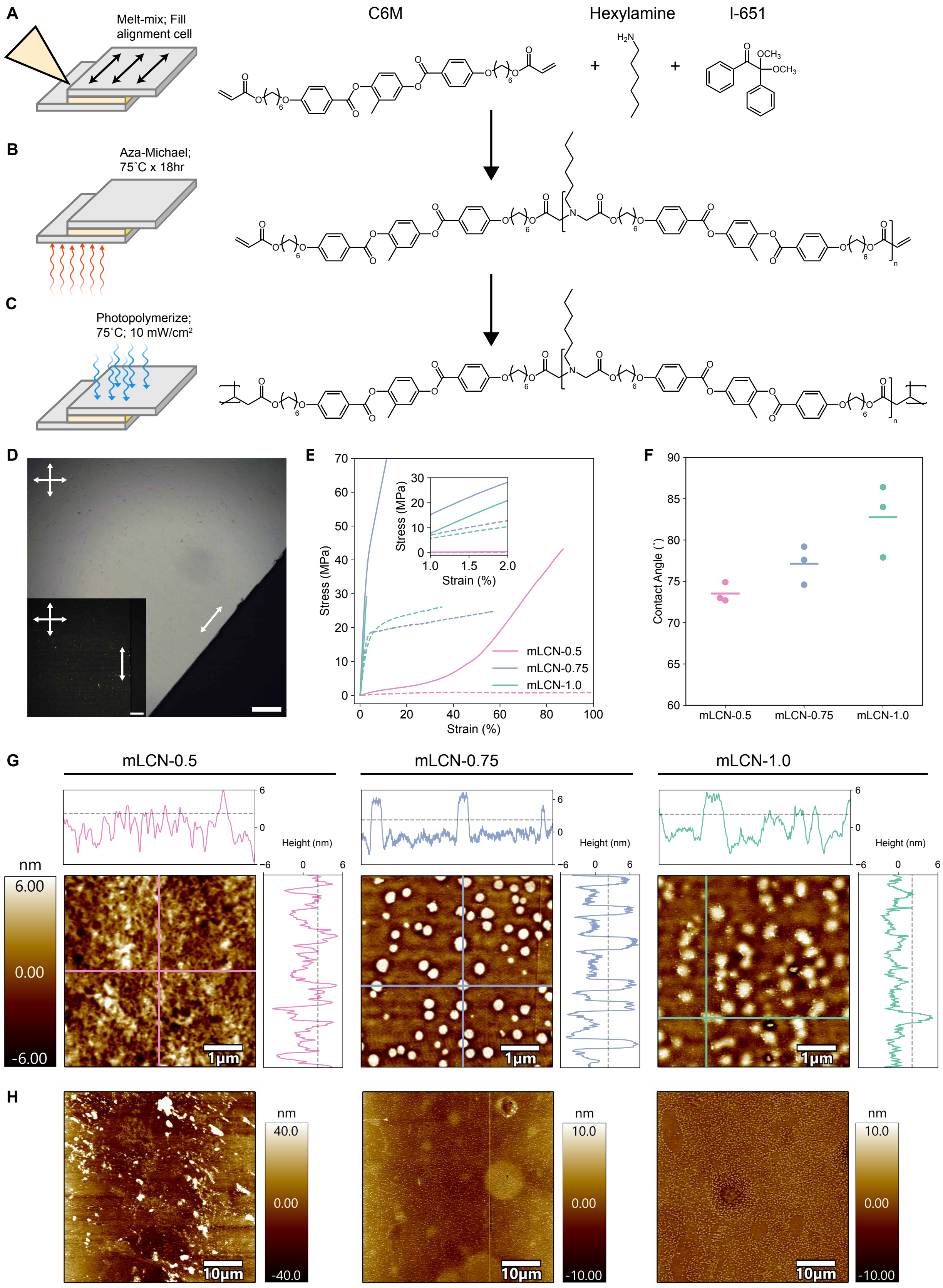
A
Melt-mix; Fill alignment cell
C6M
Hexylamine
I-651
B
Aza-Michael; 75˚C x 18hr
C
Photopolymerize; 75˚C; 10 mW/cm²
D
E
Stress (MPa)
Strain (%)
mLCN-0.5
mLCN-0.75
mLCN-1.0
F
Contact Angle (˚)
mLCN-0.5
mLCN-0.75
mLCN-1.0
G
mLCN-0.5
mLCN-0.75
mLCN-1.0
Height (nm)
nm
6.00
0.00
-6.00
1μm
H
nm
40.0
0.00
-40.0
10.0
-10.00
10μm

**Fig. S1. LCN film preparation and characterization. (A)** Liquid crystalline monomer C6M, hexylamine, and photoinitiator I-651 are melted and mixed at 85 ˚C and loaded into a glass surface alignment cell. Arrows indicate rubbing direction which corresponds to the nematic director after polymerization. **(B)** C6M reacts with hexylamine to form oligomers through aza-Michael addition reaction at 75 ˚C for 18 hr inside the cell. The molar ratio of C6M to hexylamine determines the molecular weight of the oligomers. **(C)** Acrylate-terminated oligomers are photopolymerized with UV light (10 $mW/cm^2$) for 15 minutes at 75 ˚C to form a mLCN, or at 135 ˚C to form an iLCN. **(D)** Polarized optical microscopy images demonstrate birefringence and confirm alignment of mLCN-1.0. White arrows indicate nematic director and crossed arrows indicate polarizer directions. Scale bars = 100 µm. **(E)** Representative stress-strain curves from tensile testing of mLCN-0.5, -0.75, and -1.0. Inset shows elastic region of stress-strain curves where elastic modulus values were derived. Solid lines: parallel to nematic director; dashed lines: perpendicular to nematic director. **(F)** Contact angle measurements on mLCN-0.5, mLCN-0.75, and mLCN-1.0. $N$ = 3 mLCNs per formulation; line indicates mean; all replicates shown. **(G)** High resolution AFM z maps of mLCN-0.5 (left), mLCN-0.75 (middle), and mLCN-1.0 (right) reveal surfaces with root mean square (rms) roughness ~2 nm for all samples (dashed lines in line-scan plots adjacent to images). Small, raised defects on mLCN-0.75 and mLCN-1.0 are quantified by the line-scan plots adjacent to the images and are approximately 10 nm tall and 250 nm in diameter. $N$ = 1 mLCN per formulation. Defects are randomly distributed with respect to the nematic director (horizontal axis). Scale bars = 1 µm. **(H)** Lower magnification AFM z maps of mLCN-0.5 (left), mLCN-0.75 (middle), and mLCN-1.0 (right) demonstrating the random distribution of small raised defects on mLCN-0.75 and mLCN-1.0 in relation to the nematic director (horizontal axis). Scale bars = 10 µm.

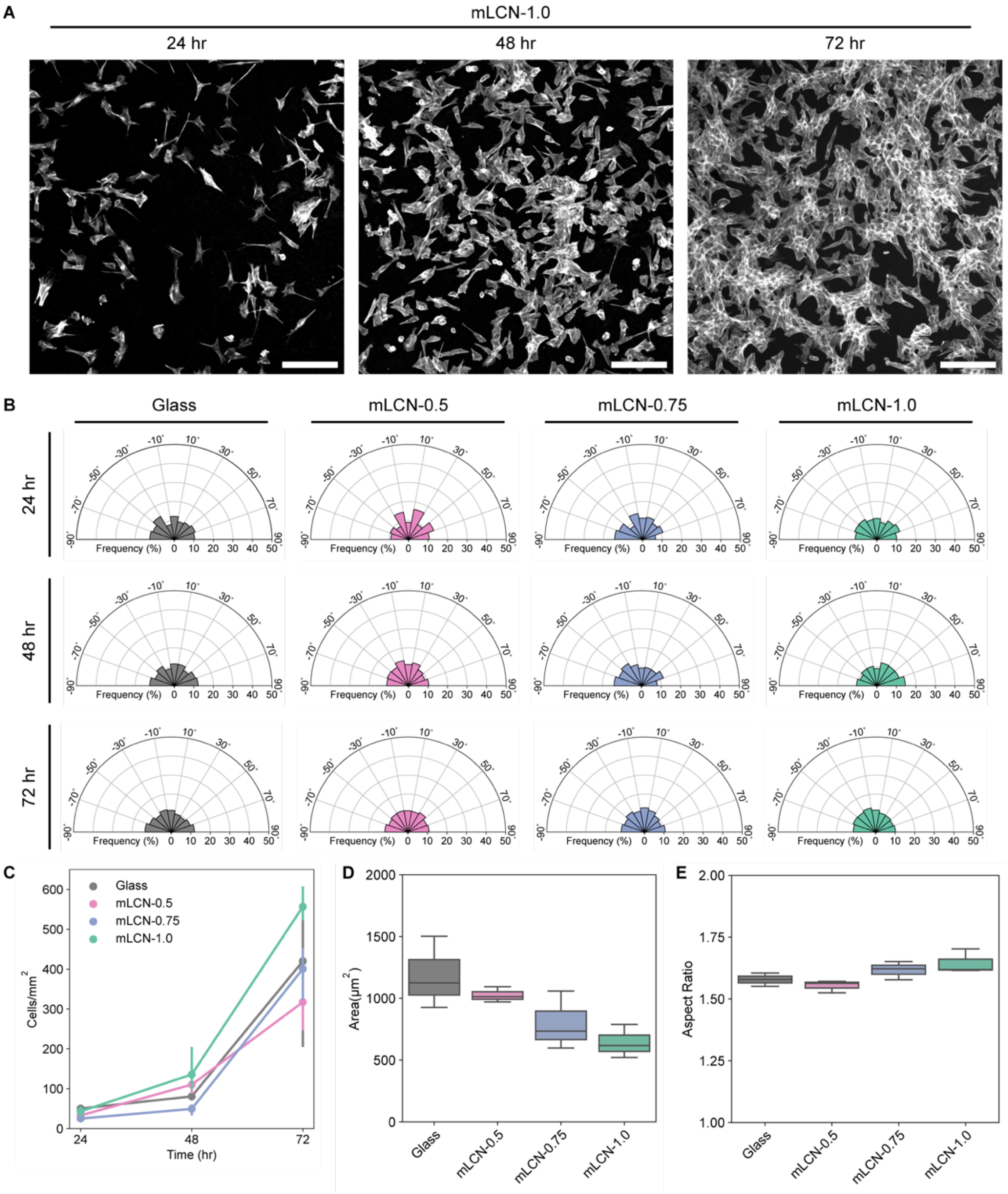


**Fig. S2. C2C12 myoblasts have no preference for the nematic director of mLCNs during initial growth phase. (A)** Representative images of C2C12 myoblasts stained with phalloidin after 24, 48, and 72 hr of growth on mLCN-1.0. Scale bars = 200 µm. **(B)** Cumulative polar histograms (20˚ bins) of C2C12 myoblast alignment for each substrate after 24, 48, and 72 hr of growth. *N* = 3 independent samples for each substrate at each timepoint; data is represented as means of binned frequencies from independent samples. **(C)** Cell density (cells/mm$^2$) for each substrate after 24, 48, and 72 hr of growth. *N* is the same as in (B); data is represented as mean ± SD. **(D)** Cell area and **(E)** aspect ratio for each substrate after 72 hr of growth. *N* is the same as in (B); box limits extend from the 25$^{th}$ to 75$^{th}$ percentiles; the horizontal line indicates the median value; the whiskers extend by 1.5x the inter-quartile range.

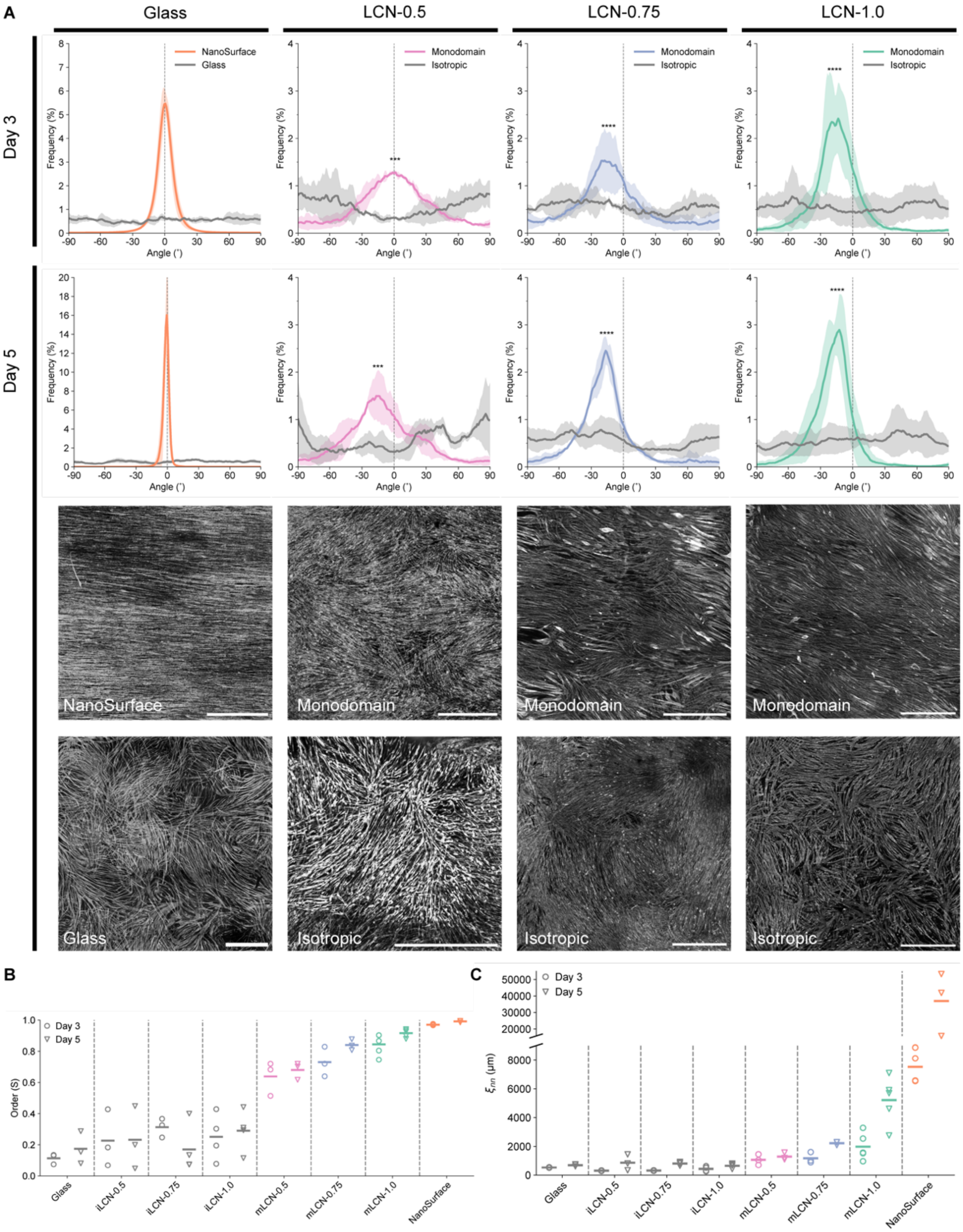

A
Glass
LCN-0.5
LCN-0.75
LCN-1.0
Day 3
Day 5
NanoSurface
Glass
Monodomain
Isotropic
Frequency (%)
Angle (˚)
***
****
NanoSurface
Monodomain
Glass
Isotropic
B
Day 3
Day 5
Order (S)
Glass
iLCN-0.5
iLCN-0.75
iLCN-1.0
mLCN-0.5
mLCN-0.75
mLCN-1.0
NanoSurface
C
Day 3
Day 5
$\xi_{nn}$ (μm)
Glass
iLCN-0.5
iLCN-0.75
iLCN-1.0
mLCN-0.5
mLCN-0.75
mLCN-1.0
NanoSurface

**Fig. S3. Myotube alignment is influenced by the degree of mLCN stiffness anisotropy. (A)** Frequency distribution plots of myotube alignment on isotropic (gray) and monodomain (colored) LCNs, and glass (gray) or NanoSurface (orange) after 3 (row 1) and 5 (row 2) days of differentiation. *N* is the same as in **Fig. 1E, F**; data are represented as mean ± SD. Statistical analysis of myotube alignment on monodomain and isotropic LCNs of the same formulation and timepoint was performed with Watson-Wheeler test for homogeneity, with significance claimed at $*P < 0.05$, $**P < 0.01$, $***P < 0.001$. $****P < 0.0001$. Row 3, 4: Representative images of myosin II heavy chain staining after 5 days of differentiation on NanoSurface, gelatin-coated glass, monodomain LCNs and isotropic LCNs. Scale bars = 1000 μm. Images of LCN-1.0 (monodomain and isotropic) are duplicated from **Fig. 1D** for completeness. **(B)** Myotube orientation-order parameter (*S*) and **(C)** nematic correlation length (μm) on all substrates after 3 and 5 days of differentiation. *N* is the same as in **Fig. 1E, F**; line indicates mean; all replicate values shown.

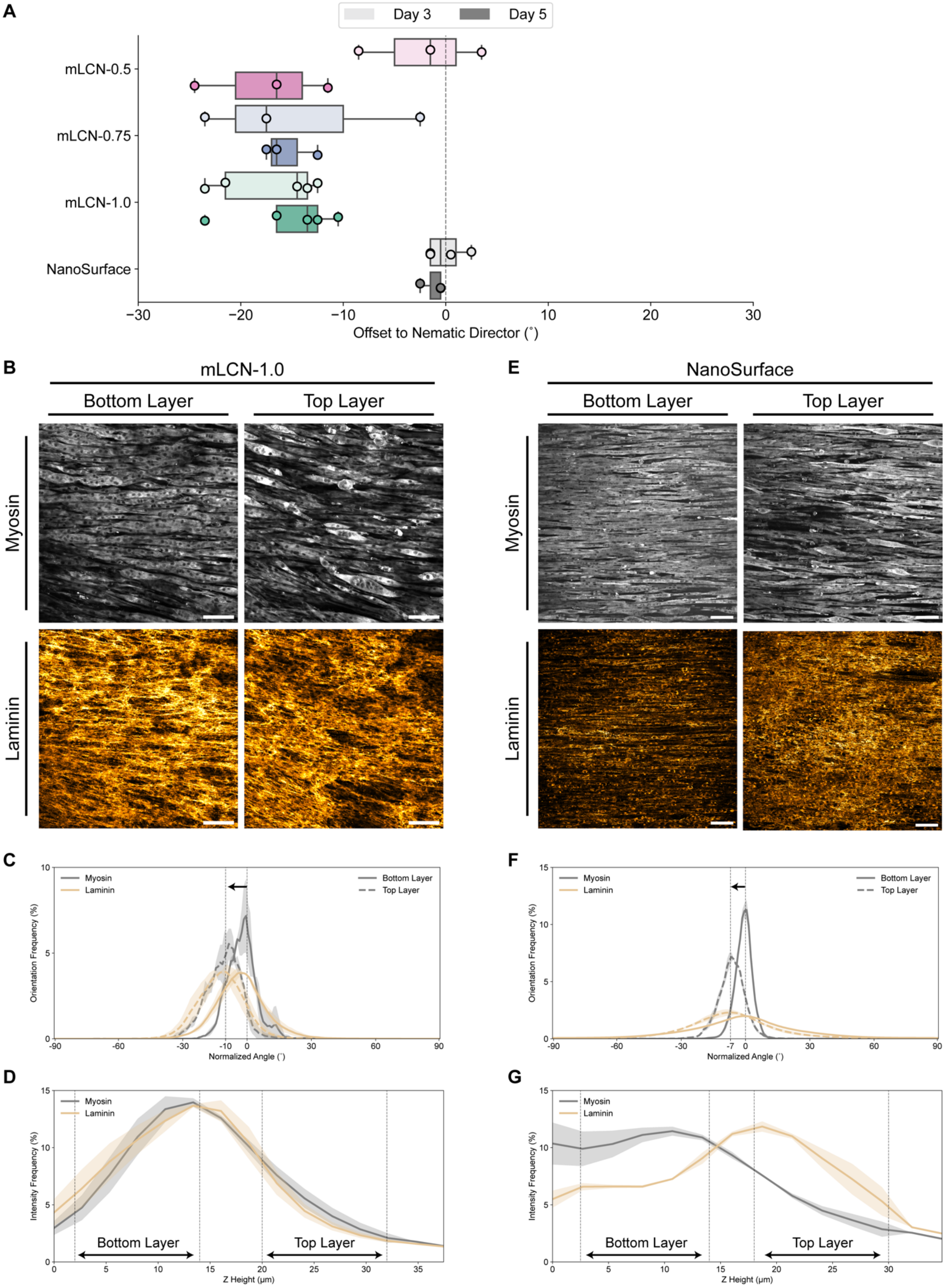
A
Day 3
Day 5
mLCN-0.5
mLCN-0.75
mLCN-1.0
NanoSurface
−30
−20
−10
0
10
20
30
Offset to Nematic Director (˚)
B
mLCN-1.0
Bottom Layer
Top Layer
Myosin
Laminin
E
NanoSurface
Bottom Layer
Top Layer
Myosin
Laminin
C
Myosin
Laminin
Bottom Layer
Top Layer
Orientation Frequency (%)
Normalized Angle (˚)
F
Myosin
Laminin
Bottom Layer
Top Layer
Orientation Frequency (%)
Normalized Angle (˚)
D
Myosin
Laminin
Intensity Frequency (%)
Bottom Layer
Top Layer
Z Height (μm)
G
Myosin
Laminin
Intensity Frequency (%)
Bottom Layer
Top Layer
Z Height (μm)

**Fig. S4. Aligned myotubes develop layers with chiral offset to the layer below. (A)** Myotube peak alignment offset to the nematic director of mLCNs and groove direction of NanoSurface substrates after 3 and 5 days of differentiation. *N* is the same as in **Fig. 1E, F**; box limits extend from the 25$^{th}$ to 75$^{th}$ percentiles; the horizontal line indicates the median value; the whiskers extend by 1.5x the inter-quartile range; all replicates including outliers shown. **(B)** Representative images of myosin II heavy chain and laminin alignment on mLCN in the bottom layer (left) and top layer (right) after 5 days of differentiation. Scale bars = 100 µm. **(C)** Corresponding orientation frequency distribution plot of myosin and laminin alignment in each layer. Arrow indicates offset from bottom layer to top layer. *N* = 2 independent samples for each substrate; data is represented as mean ± SD. **(D)** Corresponding intensity frequency distribution plot of myosin and laminin as a function of height from the bottom of the substrate. *N* = 2 independent samples for each substrate; data is represented as mean ± SD. **(E)** Representative images of myosin II heavy chain and laminin alignment on NanoSurface substrate in the bottom layer (left) and top layer (right) after 5 days of differentiation. Scale bars = 100 µm. **(F)** Corresponding orientation frequency distribution plot of myosin and laminin alignment in each layer. Arrow indicates offset from bottom layer to top layer. *N* = 2 independent samples for each substrate; data is represented as mean ± SD. **(G)** Corresponding intensity frequency distribution plot of myosin and laminin as a function of height from the bottom of the substrate. *N* = 2 independent samples for each substrate; data is represented as mean ± SD.

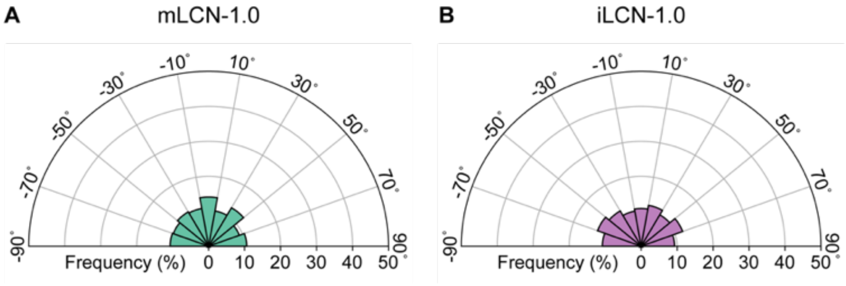


**Fig. S5. C2C12 myoblast axis of division is isotropic during proliferative phase.** Cumulative polar histograms (20˚ bins) of C2C12 myoblast division angle throughout the proliferative phase on **(A)** mLCNs and **(B)** iLCNs. *N* is the same as in **Fig. 2C, D**; data are represented as means of binned frequencies from independent samples.

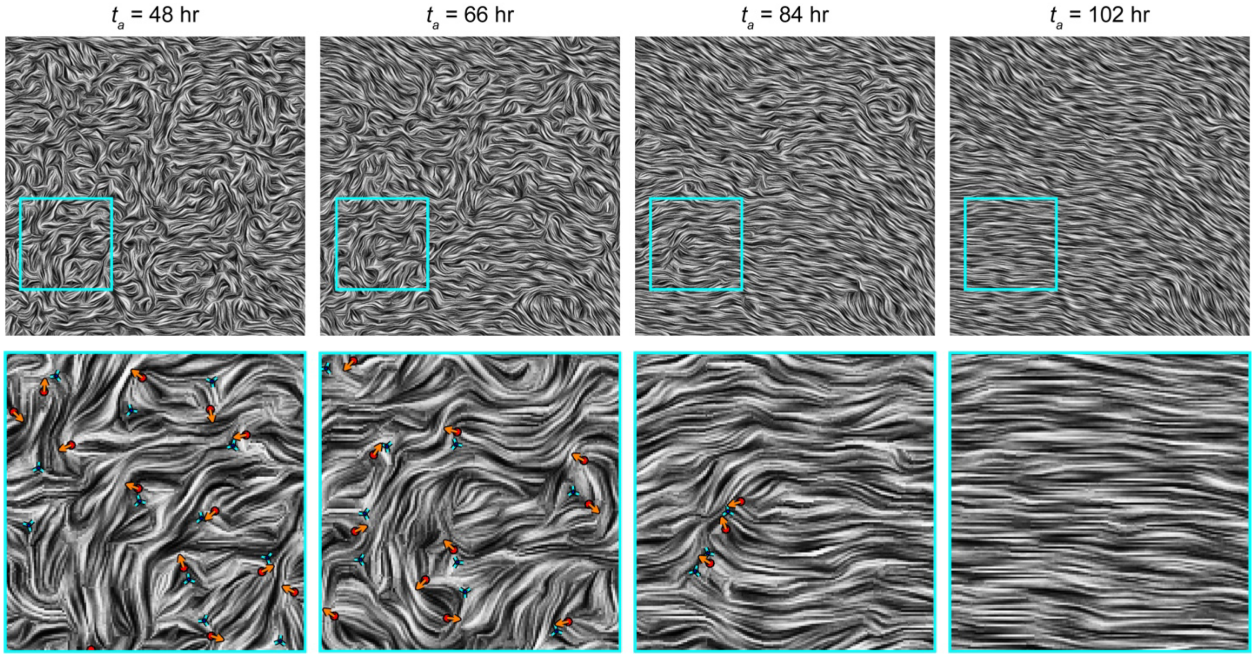


**Fig. S6. Topological defects annihilate during collective cellular flows.** Representative line integral convolution plots of myotube orientation vector field (top) and inset with topological defects labeled (bottom) on mLCNs at $t_a$ = 48, 66, 84, and 102 hr (left to right). +½ topological defects are labeled with red circles (arrow indicating orientation) and -½ topological defects are labeled with blue triangles.

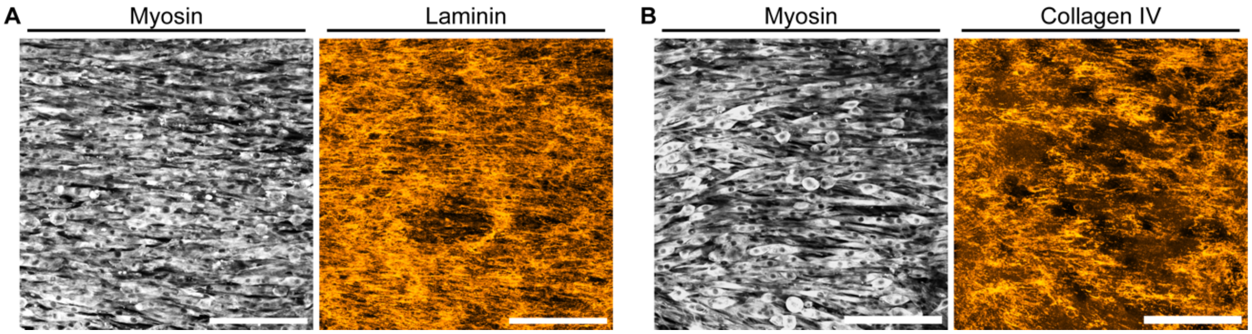


**Fig. S7. Nascent laminin and collagen IV alignment. (A)** Representative maximum intensity projections of myosin II heavy chain (left) and laminin (right) on mLCNs after 5 days of differentiation. **(B)** Representative maximum intensity projections of myosin II heavy chain (left) and collagen IV (right) on mLCNs after 5 days of differentiation. Scale bars = 200 µm.

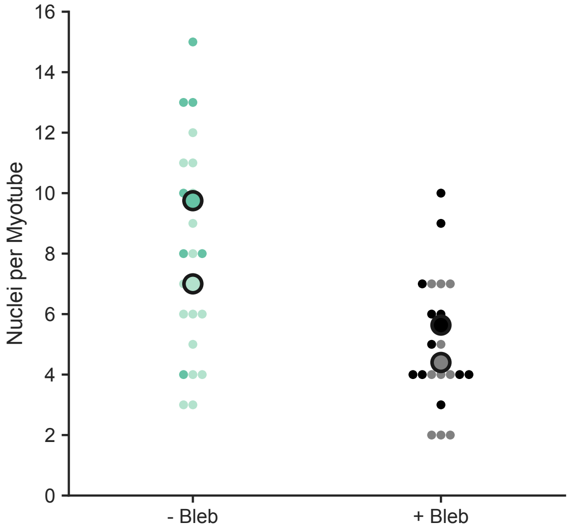


**Fig. S8. Blebbistatin treatment results in a small reduction in myotube fusion.** Distribution of nuclei per myotube after 3.5 days of differentiation on mLCNs ± blebbistatin treatment. Number of nuclei in each myotube for all replicates is displayed (N ≥ 8 myotubes per replicate); mean of each replicate is illustrated by the larger markers (N = 2 mLCNs for each condition).

| | **LCN-0.5** | **LCN-0.75** | **LCN-1.0** |
|---|---|---|---|
| **C6M (mol fraction acrylate)** | 0.5 | 0.75 | 1 |
| **Hexylamine (mol fraction amine)** | 0.5 | 0.25 | 0 |
| **I-651 (wt%)** | 2 | 2 | 2 |
| **BHT (wt%)** | 1 | 1 | 1 |

**Table S1**. **LCN chemical formulations.** See Materials and Methods for acronyms and synthesis of LCNs.

**Movie S1. Collective myoblast polarization and cellular flows on mLCNs and iLCNs.** Top: Timeseries of C2C12s on mLCN (left) and iLCN (right) stained with SiR-Actin from $t_a$ = 12–72 hr. Bottom: PIV timeseries corresponding to timeseries above. Vectors are autoscaled within each frame and overlaid on a heatmap of velocity magni`tude ranging from 0–5 μm/hr. Frames that are dimmed were excluded from PIV analysis due to stage motion artifacts.

**Movie S2. Collective cellular flows annihilate topological defects driving global myotube ordering on mLCNs.** Top left: Timeseries of C2C12s on mLCN stained with SiR-Actin from $t_a$ = 48–120 hr. Bottom left: PIV timeseries where vectors are autoscaled within each frame and overlaid on a heatmap of velocity magnitude ranging from 0–8 μm/hr. Top right: Timeseries of orientation vector field line integral convolution demonstrating annihilation of defects during collective cellular flows. Bottom right: Timeseries of local orientation-order parameter heatmap ranging from $S$ = 0–1 demonstrating local regions of disorder (black) that become ordered (white) during collective cellular flows.

**Movie S3. Inhibition of cellular contractility with blebbistatin abrogates collective cellular flows and prevents global myotube ordering.** Top: Timeseries of C2C12s on mLCN stained with SiR-Actin without (left) or with (right) addition of blebbistatin from $t_a$ = 0–84 μm/hr. Images were taken every 3 hours. Blebbistatin is first added at $t_a$ = 12 hr and media is replaced every 24 hours after. Bottom: PIV timeseries corresponding to timeseries above. Vectors are autoscaled within each frame and overlaid on a heatmap of velocity magnitude ranging from 0–7 μm/hr. Frames that are dimmed were excluded from PIV analysis due to stage motion artifacts.

## REFERENCES AND NOTES


1. T. Panciera, L. Azzolin, M. Cordenonsi, S. Piccolo, Mechanobiology of YAP and TAZ in physiology and disease. *Nat. Rev. Mol. Cell Biol.* **18**, 758–770 (2017).

2. K. H. Vining, D. J. Mooney, Mechanical forces direct stem cell behaviour in development and regeneration. *Nat. Rev. Mol. Cell Biol.* **18**, 728–742 (2017).

3. C. M. Lo, H. B. Wang, M. Dembo, Y. L. Wang, Cell movement is guided by the rigidity of the substrate. *Biophys. J.* **79**, 144–152 (2000).

4. R. Sunyer, V. Conte, J. Escribano, A. Elosegui-Artola, A. Labernadie, L. Valon, D. Navajas, J. M. Garcia-Aznar, J. J. Munoz, P. Roca-Cusachs, X. Trepat, Collective cell durotaxis emerges from long-range intercellular force transmission. *Science* **353**, 1157–1161 (2016).

5. A. Shellard, R. Mayor, Collective durotaxis along a self-generated stiffness gradient in vivo. *Nature* **600**, 690–694 (2021).

6. I. B. Bischofs, U. S. Schwarz, Cell organization in soft media due to active mechanosensing. *Proc. Natl. Acad. Sci. U.S.A.* **100**, 9274–9279 (2003).

7. A. Saez, M. Ghibaudo, A. Buguin, P. Silberzan, B. Ladoux, Rigidity-driven growth and migration of epithelial cells on microstructured anisotropic substrates. *Proc. Natl. Acad. Sci. U.S.A.* **104**, 8281–8286 (2007).

8. A. Islam, M. Younesi, T. Mbimba, O. Akkus, Collagen substrate stiffness anisotropy affects cellular elongation, nuclear shape, and stem cell fate toward anisotropic tissue lineage. *Adv. Healthc. Mater.* **5**, 2237–2247 (2016).

9. K. M. Herbert, H. E. Fowler, K. R. Schlafmann, J. A. Koch, T. J. White, Synthesis and alignment of liquid crystalline elastomers. *Nat. Rev. Mater.* **7**, 23–38 (2022).

10. T. H. Ware, T. J. White, Programmed liquid crystal elastomers with tunable actuation strain. *Polym. Chem.* **6**, 4835–4844 (2015).

11. C. M. Yakacki, M. Saed, D. P. Nair, T. Gong, S. M. Reed, C. N. Bowman, Tailorable and programmable liquid-crystalline elastomers using a two-stage thiol-acrylate reaction. *RSC Adv.* **5**, 18997–19001 (2015).

12. T. H. Ware, M. E. McConney, J. J. Wie, V. P. Tondiglia, T. J. White, Voxelated liquid crystal elastomers. *Science* **347**, 982–984 (2015).

13. T. Turiv, J. Krieger, G. Babakhanova, H. Yu, S. V. Shiyanovskii, Q. Wei, M. Kim, O. D. Lavrentovich, Topology control of human cells monolayer by liquid crystal elastomer. *Sci. Adv.* **651**, 1–11 (2020).

14. H. Finkelmann, J. Benné, K. Semmler, Nematic liquid single crystal elastomers. *Makromol. Chem. Rapid Commun.* **12**, 717–726 (1991).

15. Y. Luo, M. Gu, M. Park, X. Fang, Y. Kwon, J. M. Uruena, J. Read de Alaniz, M. E. Helgeson, M. C. Marchetti, M. T. Valentine. Molecular-scale substrate anisotropy and crowding drive long-range nematic order of cell monolayers. arXiv:2210.13425 [physics.bio-ph] (2022).

16. D. Martella, L. Pattelli, C. Matassini, F. Ridi, M. Bonini, P. Paoli, P. Baglioni, D. S. Wiersma, C. Parmeggiani, Liquid crystal-induced myoblast alignment. *Adv. Healthc. Mater.* **8**, 1–10 (2019).

17. D. A. Morrow, T. L. H. Donahue, G. M. Odegard, K. R. Kaufman, Transversely isotropic tensile material properties of skeletal muscle tissue. *J. Mech. Behav. Biomed. Mater.* **3**, 124–129 (2010).

18. J. P. K. Armstrong, J. L. Puetzer, A. Serio, A. G. Guex, M. Kapnisi, A. Breant, Y. Zong, V. Assal, S. C. Skaalure, O. King, T. Murty, C. Meinert, A. C. Franklin, P. G. Bassindale, M. K. Nichols, C. M. Terracciano, D. W. Hutmacher, B. W. Drinkwater, T. J. Klein, A. W. Perriman, M. M. Engineering anisotropic muscle tissue using acoustic cell patterning. *Adv. Mater.* **30**, e1802649 (2018).

19. J. R. Sanes, The basement membrane/basal lamina of skeletal muscle. *J. Biol. Chem.* **278**, 12601–12604 (2003).

20. R. Vracko, E. P. Benditt, Basal lamina: The scaffold for orderly cell replacement: Observations on regeneration of injured skeletal muscle fibers and capillaries. *J. Cell Biol.* **55**, 406–419 (1972).

21. P. A. Pranda, A. Hedegaard, H. Kim, J. Clapper, E. Nelson, L. Hines, R. C. Hayward, T. J. White, Directional adhesion of monodomain liquid crystalline elastomers. *ACS Appl. Mater. Interfaces* **16**, 6394–6402 (2024).

22. W. A. Loesberg, J. te Riet, F. C. M. J. M. van Delft, P. Schon, C. G. Figdor, S. Speller, J. J. W. A. van Loon, X. F. Walboomers, J. A. Jansen, The threshold at which substrate nanogroove dimensions may influence fibroblast alignment and adhesion. *Biomaterials* **28**, 3944–3951 (2007).

23. E. Lamers, X. F. Walboomers, M. Domanski, J. te Riet, F. C. M. J. M. van Delft, R. Luttge, L. A. J. A. Winnubst, H. J. G. E. Gardeniers, J. A. Jansen, The influence of nanoscale grooved substrates on osteoblast behavior and extracellular matrix deposition. *Biomaterials* **31**, 3307–3316 (2010).

24. Q. Mao, A. Acharya, A. Rodriguez-delaRosa, F. Marchiano, B. Dehapiot, Z. A. Tanoury, J. Rao, M. Diaz-Cuadros, A. Mansur, E. Wagner, C. Chardes, V. Gupta, P. Lenne, B. H. Habermann, O. Theodoly, O. Pourquie, F. Schnorrer, Tension-driven multi-scale self-organisation in human iPSC-derived muscle fibers. *eLife* **11**, e76649 (2022).

25. R. A. Latour, Biomaterials: Protein–surface interactions, in *Encyclopedia of Biomaterials and Biomedical Engineering* (CRC Press, ed. 2, 2008), pp. 1–15.

26. D. Yaffe, S. Ora, Serial passaging and differentiation of myogenic cells isolated from dystrophic mouse muscle. *Nature* **270**, 725–727 (1977).

27. S. M. Abmayr, G. K. Pavlath, Myoblast fusion: Lessons from flies and mice. *Development* **139**, 641–656 (2012).

28. S. Burattini, P. Ferri, M. Battistelli, R. Curci, F. Luchetti, E. Falcieri, C2C12 murine myoblasts as a model of skeletal muscle development: Morpho-functional characterization. *Eur. J. Histochem.* **48**, 223–233 (2004).

29. R. Rezakhaniha, A. Agianniotis, J. T. C. Schrauwen, A. Griffa, D. Sage, C. V. C. Bouten, F. N. van de Vosse, M. Unser, N. Stergiopulos, Experimental investigation of collagen waviness and orientation in the arterial adventitia using confocal laser scanning microscopy. *Biomech. Model. Mechanobiol.* **11**, 461–473 (2012).

30. D. Mistry, H. F. Gleeson, Mechanical deformations of a liquid crystal elastomer at director angles between 0° and 90°: Deducing an empirical model encompassing anisotropic nonlinearity. *J. Polym. Sci. Part B Polym. Phys.* **57**, 1367–1377 (2019).

31. S. Okamoto, S. Sakurai, K. Urayama, Effect of stretching angle on the stress plateau behavior of main-chain liquid crystal elastomers. *Soft Matter* **17**, 3128–3136 (2021).

32. S. Garcia, E. Hannezo, J. Elgeti, J. Joanny, P. Silberzan, N. S. Gov, Physics of active jamming during collective cellular motion in a monolayer. *Proc. Natl. Acad. Sci. U.S.A.* **112**, 15314–15319 (2015).

33. T. Sarkar, V. Yashunsky, L. Brezin, C. B. Mercader, T. Aryaksama, M. Lacroix, T. Risler, J. Joanny, P. Silberzan, Crisscross multilayering of cell sheets. *PNAS Nexus* **2**, 1–11 (2023).

34. Y. Sun, R. Duffy, A. Lee, A. W. Feinberg, Optimizing the structure and contractility of engineered skeletal muscle thin films. *Acta Biomater.* **9**, 7885–7894 (2013).

35. W. Zhang, Y. Liu, H. Zhang, Extracellular matrix: An important regulator of cell functions and skeletal muscle development. *Cell Biosci.* **11**, 1–13 (2021).

36. M. J. Mondrinos, F. Alisafaei, A. Y. Yi, H. Ahmadzadeh, I. Lee, C. Blundell, J. Seo, M. Osborn, T. Jeon, S. M. Kim, V. B. Shenoy, D. Huh, Surface-directed engineering of tissue anisotropy in microphysiological models of musculoskeletal tissue. *Sci. Adv.* **7**, eabe9446 (2021).

37. F. Alisafaei, D. Shakiba, L. E. Iannucci, M. D. Davidson, K. M. Pryse, P. G. Chao, J. A. Burdick, S. P. Lake, E. L. Elson, V. B. Shenoy, G. M. Genin, Tension anisotropy drives phenotypic transitions of cells via two-way cell-ECM feedback. bioRxiv 2022.03.13.484154 [Preprint] (2022). https://doi.org/10.1101/2022.03.13.484154.

38. K. H. Palmquist, S. F. Tiemann, F. L. Ezzeddine, S. Yang, C. R. Pfeifer, A. Erzberger, A. R. Rodrigues, A. E. Shyer, Reciprocal cell-ECM dynamics generate supracellular fluidity underlying spontaneous follicle patterning. *Cell* **185**, 1960–1973.e11 (2022).

39. C. Bruyère, M. Versaeval, D. Mohammed, L. Alaimo, M. Luciano, E. Vercruysse, S. Gabriele, Actomyosin contractility scales with myoblast elongation and enhances differentiation through YAP

nuclear export. *Sci. Rep.* **9**, 1–14 (2019).

40. J. Kim, J. Feng, C. A. R. Jones, X. Mao, L. M. Sander, H. Levine, B. Sun, Stress-induced plasticity of dynamic collagen networks. *Nat. Commun.* **8**, 842 (2017).

41. J. d'Alessandro, A. Barbier-Chebbah, V. Cellerin, O. Benichou, R. M. Mege, R. Voituriez, B. Ladoux, Cell migration guided by long-lived spatial memory. *Nat. Commun.* **12**, 4118 (2021).

42. F. M. Yavitt, B. E. Kirkpatrick, M. R. Blatchley, K. F. Speckl, E. Mohagheghian, R. Moldovan, N. Wang, P. J. Dempsey, K. S. Anseth, In situ modulation of intestinal organoid epithelial curvature through photoinduced viscoelasticity directs crypt morphogenesis. *Sci. Adv.* **9**, eadd5668 (2023).

43. G. Thrivikraman, A. Jagiello, V. K. Lai, S. L. Johnson, M. Keating, A. Nelson, B. Schultz, C. M. Wang, A. J. Levine, E. L. Botvinick, R. T. Tranquillo, Cell contact guidance via sensing anisotropy of network mechanical resistance. *Proc. Natl. Acad. Sci. U.S.A.* **118**, 1–11 (2021).

44. C. Obbink-Huizer, C. W. J. Oomens, S. Loerakker, J. Foolen, C. V. C. Bouten, F. P. T. Baaijens, Computational model predicts cell orientation in response to a range of mechanical stimuli. *Biomech. Model. Mechanobiol.* **13**, 227–236 (2014).

45. T. E. Angelini, E. Hannezo, X. Trepat, M. Marquez, J. J. Fredberg, D. A, Weitz, Glass-like dynamics of collective cell migration. *Proc. Natl. Acad. Sci. U.S.A.* **108**, 4714–4719 (2011).

46. T. B. Saw, W. Xi, B. Ladoux, C. T. Lim, Biological tissues as active nematic liquid crystals. *Adv. Healthc. Mater.* **30**, e1802579 (2018).

47. P. Guillamat, C. Blanch-Mercader, G. Pernollet, K. Kruse, A. Roux, Integer topological defects organize stresses driving tissue morphogenesis. *Nat. Mater.* **21**, 588–597 (2022).

48. R. Thiagarajan, A. Bhat, G. Salbreux, M. M. Inamdar, D. Riveline, Pulsations and flows in tissues as two collective dynamics with simple cellular rules. *iScience* **25**, 105053 (2022).

49. L. Balasubramaniam, A. Doostmohammadi, T. B. Saw, G. H. N. S. Narayana, R. Mueller, T. Dang, M. Thomas, S. Gupta, S. Sonam, A. S. Yap, Y. Toyama, R. Mege, J. M. Yeomans, B. Ladoux, Investigating

the nature of active forces in tissues reveals how contractile cells can form extensile monolayers. *Nat. Mater.* **20**, 1156–1166 (2021).

50. I. Batalov, Q. Jallerat, S. Kim, J. Bliley, A. W. Feinberg, Engineering aligned human cardiac muscle using developmentally inspired fibronectin micropatterns. *Sci. Rep.* **11**, 11502 (2021).

51. C. R. Pfeifer, A. E. Shyer, A. R. Rodrigues, Creative processes during vertebrate organ morphogenesis: Biophysical self-organization at the supracellular scale. *Curr. Opin. Cell Biol.* **86**, 102305 (2024).

52. A. E. Shyer, A. R. Rodrigues, G. G. Schroeder, E. Kassianidou, S. Kumar, R. M. Harland, Emergent cellular self-organization and mechanosensation initiate follicle pattern in the avian skin. *Science* **357**, 811–815 (2017).

53. S. Yang, K. H. Palmquist, L. Nathan, C. R. Pfeifer, P. J. Schultheiss, A. Sharma, L. C. Kam, P. W. Miller, A. E. Shyer, A. R. Rodrigues, Morphogens enable interacting supracellular phases that generate organ architecture. *Science* **382**, eadg5579 (2023).

54. A. J. Engler, M. A. Griffin, S. Sen, C. G. Bonnemann, H. L. Sweeney, D. E. Discher, Myotubes differentiate optimally on substrates with tissue-like stiffness: Pathological implications for soft or stiff microenvironments. *J. Cell Biol.* **166**, 877–887 (2004).

55. U. Schmidt, M. Weigert, C. Broaddus, G. Myers, Cell detection with star-convex polygons. arXiv:1806.03535 [cs.CV] (2018).

56. M. Weigert, U. Schmidt, R. Haase, K. Sugawara, G. Myers, Star-convex polyhedra for 3D object detection and segmentation in microscopy. arXiv:1908.03636 [cs.CV] (2019).

57. O. J. Meacock, A. Doostmohammadi, K. R. Foster, J. M. Yeomans, W. M. Durham, Bacteria solve the problem of crowding by moving slowly. *Nat. Phys.* **17**, 205–210 (2021).

58. A. Umeno, H. Kotani, M. Iwasaka, S. Ueno, Quantification of adherent cell orientation and morphology under strong magnetic fields. *IEEE Trans. Magn.* **37**, 2909–2911 (2001).

59. M. D. Davidson, M. E. Prendergast, E. Ban, K. L. Xu, G. Mickel, P. Mensah, A. Dhand, P. A. Janmey, V. B. Shenoy, J. A. Burdick, Programmable and contractile materials through cell encapsulation in fibrous hydrogel assemblies. *Sci. Adv.* **7**, eabi8157 (2021).

60. L. Q. Wan, K. Ronaldson, M. Park, G. Taylor, Y. Zhang, F. M. Gimble, G. Vunjak-Novakovic, Micropatterned mammalian cells exhibit phenotype-specific left-right asymmetry. *Proc. Natl. Acad. Sci. U.S.A.* **108**, 12295–12300 (2011).

61. T. Rahman, F. Peters, L. Q. Wan, Cell jamming regulates epithelial chiral morphogenesis. *J. Biomech.* **147**, 111435 (2023).

62. G. Duclos, C. Blanch-Mercader, V. Yashunsky, G. Salbreux, J. F. Joanny, J. Prost, P. Silberzan, Spontaneous shear flow in confined cellular nematics. *Nat. Phys.* **14**, 728–732 (2018).
